# Human primary antibody response to vaccination follows a partially sequential class-switching program with a checkpoint at *IGHG2*

**DOI:** 10.1016/j.xcrm.2026.102848

**Published:** 2026-06-04

**Authors:** Guillem Montamat-Garcia, Joseph C.F. Ng, Alexander T. Stewart, Emma Sinclair, Benedicta B. Mensah, Yan Hui Giam, Paul Blair, Diana Kateregga, Amir Gander, David Kipling, Dongjun Guo, Lutecia Servius, Christopher J.M. Piper, Zara Baig, Franca Fraternali, Claudia Mauri, Deborah K. Dunn-Walters

**Affiliations:** 1 Institute of Immunity and Transplantation, Division of Infection and Immunity, Royal Free Hospital, University College London, London NW3 2PP, UK; 2 Research Department of Structural and Molecular Biology, Division of Biosciences & Institute of Structural and Molecular Biology, University College London, Gower Street, London WC1E 6BT, UK; 3 Department of Biological Sciences, Birkbeck, University of London, London WC1E 7HX, UK; 4 School of Biosciences, University of Surrey, Guildford GU2 7XH, UK; 5 Animal and Plant Health Agency, Addlestone, Surrey KT15 3NB, UK; 6 Department of Immunobiology, School of Immunology and Microbial Sciences, Kings College London, London SE1 1UL, UK; 7 Tissue Access for Patient Benefit, Royal Free Hospital, University College London, London NW3 2PP, UK

**Keywords:** antibody response, class switch recombination, CSR, vaccination response, human antibody class switching, B cell subtypes

## Abstract

Class-switch recombination (CSR) allows B cells to produce antibodies with distinct effector functions, but its dynamics during a primary human response remain poorly understood. We sampled COVID-19-naive healthy volunteers every other day during the first 3 weeks after SARS-CoV-2 vaccination, combining bulk and single-cell B cell receptor repertoires, single-cell transcriptomics, immunophenotyping, and *IGHC* sterile transcript analysis. Vaccine-specific B cells show sterile transcription across all *IGHC* genes up to *IGHG2*, contradicting the prevailing idea of single-gene sterile transcription. Clonal tracking confirms that sequential CSR exists: e.g., *IGHG3* to *IGHG1* and *IGHG1* to *IGHA1* and *IGHG2*, with sparse switching beyond *IGHG2*. VDJ gene usage associates with specific isotype subclasses and differential CSR timing. CSR and somatic hypermutation are temporally decoupled, with antigen-specific clones remaining hypomutated up to 10 weeks post-immunization. These findings complement textbook models of CSR and inform strategies for vaccines requiring switching to key isotypes such as IgG1 or IgA2.

## Highlights

• SARS-CoV-2 vaccination induces sequential class switch recombination up to *IGHG2*
• *IGHC* sterile transcription depends on the B cell subset during SARS-CoV-2 vaccination
• VDJ gene usage associates with differential CSR timing during SARS-CoV-2
• Class-switching unlinks from somatic hypermutation during SARS-CoV-2 vaccination

## Graphical abstract

**Figure:**
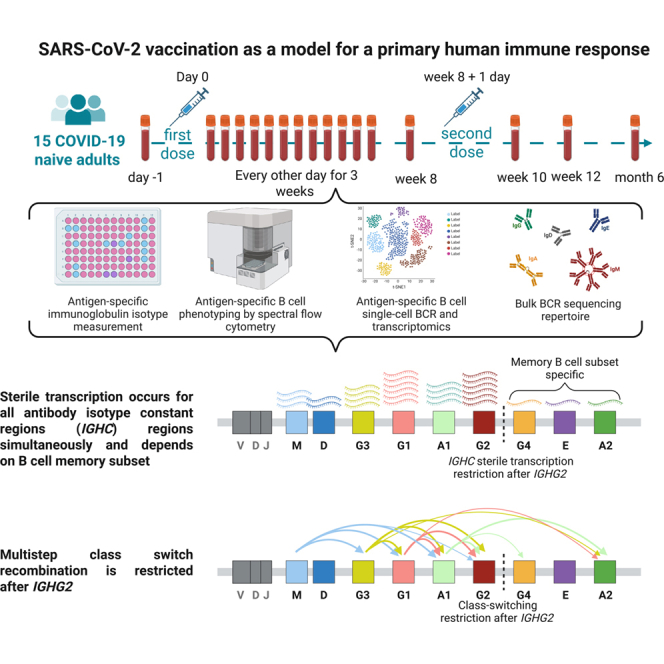

## Teaser

Using the SARS-CoV-2 mRNA vaccine as a model immune challenge, Montamat-Garcia et al. describe how antibody class-switch recombination (CSR) during a primary human response is partially sequential, but with checkpoint at the *IGHG2* locus. Moreover, CSR is influenced by the B cell subset and VDJ gene usage.

## Introduction

B cells, the mainstay of humoral responses, exist in many different developmental and functional forms, including regulators, antigen presenters, T cell interactors, as well as antibody producers. Since B cells are very often underrepresented and poorly analyzed in immune single-cell RNA sequencing (scRNA-seq) data, public cell atlases are lacking in crucial B cell information, especially during primary immune responses. B cells confer specificity against immune challenges via immunoglobulins (Ig), which can be either membrane bound (B cell receptor [BCR]) or secreted in the form of antibodies.^1^ In humans, there are 5 isotypes, or classes, of BCRs with subtypes for IgG and IgA (IgM, IgD, IgE, IgG1/2/3/4, and IgA1/2).^2^ Identifying specific BCR isotypes is important as they are linked to distinct immune functions and responses and antibody-derived diseases. For example, IgA is predominantly localized to mucosal surfaces, including those in the respiratory and gastrointestinal tracts,^3^^,^^4^^,^^5^ while IgG, the most abundant antibody isotype in serum, plays a key role in immune responses against viral infections.^6^^,^^7^^,^^8^ The subtype of a BCR can also show important effector and functional differences, i.e., IgG1 and IgG2 have different Fc receptor-binding affinities^9^^,^^10^ and IgA2 can dimerize and be secreted across mucosal surfaces, while IgA1 remains mainly systemic.^11^ Particular antibody isotypes are also associated with diseases, for instance, IgA with IgA nephropathy, IgE as a hallmark of allergic reactions, and specific IgG subtypes with autoimmunity.^12^^,^^13^^,^^14^^,^^15^

Distinguishing between subtypes in high-resolution, single-cell settings has been difficult in immunology, but newer genomic and transcriptomic techniques can provide information on the type of B cell,^16^ its maturity in a response,^17^ and fundamental B cell processes such as class-switch recombination (CSR).^18^ This new information can provide key insights into the role of B cells in disease conditions such as autoimmunity or responses to antigenic challenges of infection and vaccination.^19^^,^^20^^,^^21^^,^^22^

During CSR, B cells switch their BCR subtypes by undergoing irreversible DNA recombination at their immunoglobulin heavy-chain (*IGH*) loci,^23^^,^^24^^,^^25^ in order to bring a different constant region gene (*IGHC*) into proximity with the variable region to form a productive Ig transcript. The irreversibility of these events, due to genomic deletion, imposes a 5′ to 3′ directionality on CSR.^23^^,^^24^ The transcriptional events that open the genomic DNA to cutting and splicing factors during CSR result in the production of non-coding, “sterile” *IGHC* transcripts. Sterile transcripts have often been neglected due to their lack of contribution to the productive Ig output of B cells. Although the presence of sterile transcripts alone is insufficient to guarantee CSR, as evidenced in studies of sterile transcription-reporter mice,^26^ sterile transcripts indicate the propensity of B cells poised to CSR^27^ and therefore, serve as markers to predict the likely future CSR directionality.^26^^,^^28^ Our recent development of dedicated computational pipelines^18^ has facilitated the study of CSR directionality preferences with sterile transcripts, using scRNA-seq data.

Despite advancements in the study of CSR, a knowledge gap exists regarding the role of human B cell CSR during primary immune responses, particularly in understanding the dynamics of this process and potential preferential pathways to specific final *IGHC* when naive individuals encounter an antigen for the first time. It has long been debated whether CSR occurs directly (exclusively from IgM-expressing naive B cells), indirectly (via intermediate *IGHC* genes), or as a combination of the two.^23^^,^^24^^,^^29^ Most adult human immune response studies on vaccinated individuals are limited in longitudinal follow-up and involve vaccine challenges within the geography of endemic disease, which means they will likely be a secondary challenge to a previous primary exposure. Although animal models allowed the characterization of CSR in a primary immune response,^30^ there are substantial differences in the genomic architecture of the *IGHC* locus between mice and humans.^31^^,^^32^^,^^33^

Here, we took advantage of an antigen-naive cohort being vaccinated with the SARS-CoV-2 mRNA-1273 vaccine to obtain detailed multi-omic B cell data from samples taken at frequent time points post-vaccination. These primary response data include bulk and single-cell BCR sequencing, single-cell transcriptomics, and flow cytometry immune phenotyping of whole blood and isolated lymphocytes along with saliva samples collected under a post-vaccination (first and second doses) strict schedule, as well as at a 6-month post-immunization time point. This allowed us to finely map B cell, CSR and antibody production dynamics during primary immunization in the context of vaccine-derived antigen-specific B cells. Data from this multi-omic resource can be visualized in an integrated and interactive web browser (https://fraternalilab.cs.ucl.ac.uk/CovVaxBcells/).

In contrast to a previous report, in which CSR dynamics was inferred under homeostatic conditions at a single time point,^34^ we show that over a detailed time course of several weeks CSR follows a partially sequential pattern. This pattern is characterized by a predominant switching preference to adjacent *IGHC* regions and a checkpoint at *IGHG2*. This resulted in high IgG1 but low IgA (both IgA1 and IgA2) expression by antigen-specific B cells, which could explain the strong protection against severe COVID-19 complications but the limited mucosal immunity^35^^,^^36^ and consequently, the reduced ability of mRNA vaccines against SARS-CoV-2 to block transmission. Newly activated B cells produce sterile transcripts from all *IGHC* regions up to *IGHG2*. However, some subsets of memory B cells can express sterile transcripts beyond this point. This changes the textbook understanding of CSR dynamics and is important knowledge for the manipulation of CSR outcomes, i.e., stopping CSR from continuing beyond *IGHG1*, or promoting CSR beyond *IGHG2* to *IGHA2*, with the aim of improving vaccine design and developing therapeutic interventions in antibody-based diseases.

## Results

### Antigen-specific, class-switched, CD27-negative memory B cells appear after the second dose of SARS-CoV-2 mRNA vaccine and remain long term

**Figure 1:**
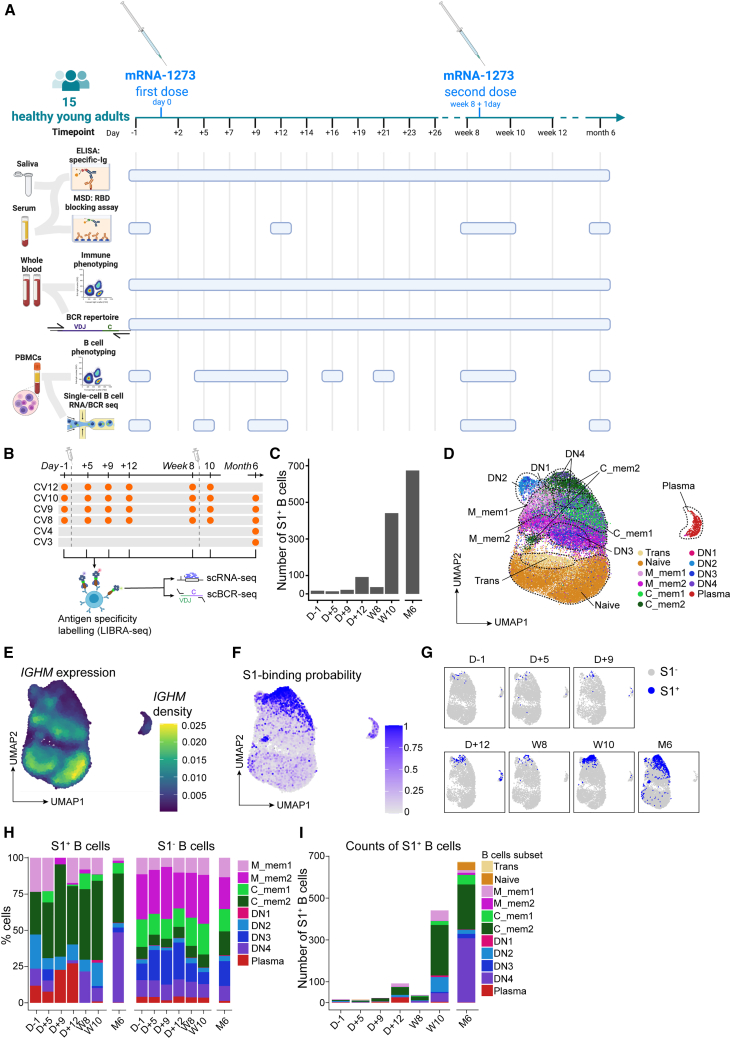
Multi-immunomic SARS-CoV-2 vaccination study detects antigen-specific class-switched double-negative (DN) memory B cells after the second dose (A) Schematic illustrating sample collection timeline and assays performed on the collected samples. The schedule included a baseline on day −1, with initial vaccination on day 0, followed by time points every Monday, Wednesday, and Friday from days 2 to 26. The second dose was administered at week 8, with pre-dose baseline (W8) and post-dose time points at weeks 10 and 12, concluding with a final check at 6 months post-initial vaccination. (B) Schematic illustration of time points selected for single-cell transcriptomic profiling. *n* = 4 (from D−1 to W10) or 5 (M6) for the rest of the figure. (C) Number of S1^+^ B cells captured in single-cell transcriptomic data across time points. (D) Projection of scRNA-seq data of *n* = 35,426 B cells (S1^+^ and S1^−^ B cells) using uniform manifold approximation and projection (UMAP). Cell labels are transferred from a previously published scRNA-seq atlas of peripheral B cells based on gene expression similarities (see STAR Methods for details). Trans, transitional B cells; M_mem, IgM memory B cells; C_mem, classical memory B cells, DN, double-negative B cells; plasma, plasmablasts. (E) *IGHM* gene expression per cell in the scRNA-seq data and visualized on the UMAP projection. (F) S1-binding probability score quantified per cell in the scRNA-seq data and visualized on the UMAP projection. (G) S1^+^ (blue) and S1^−^ (gray) B cells at each time point assayed in scRNA-seq visualized using the UMAP projection overtime. (H) Relative frequency distribution of S1^+^ and S1^−^ memory B cells and plasma cells across time; *n* = 4/5. (I) Number of S1^+^ B cells sampled at each time point grouped by B cell subset labels; *n* = 4/5.

We took advantage of the SARS-CoV-2 mRNA-1273 vaccination as a model to address the knowledge gap in human CSR dynamics and B cell clonal evolution during primary responses. Fifteen healthy adults were immunized with the SARS-CoV-2 mRNA-1273 vaccine and samples collected for multi-omic measurement using a comprehensive schedule with time points every other day for the first 3 weeks (details in STAR Methods and Figure 1A). Two (P6 and P14) of 15 participants were excluded after testing positive for anti-receptor-binding domain (RBD) of the SARS-CoV-2 Spike (S) protein IgG antibodies, indicating prior SARS-CoV-2 infection (Figure S1A).

Vaccine-induced RBD-specific IgG, IgA, and IgM antibodies emerged in the serum by day 12 post-vaccination (D+12), with IgG increasing significantly after the second dose, while IgA and IgM remaining unchanged; unlike in infection, salivary IgA did not rise during vaccination (Figures S1B and S1C). By month 6 (M6), all RBD-specific antibodies declined, although sera-blocking capacity against multiple SARS-CoV-2 strains persisted, except against Omicron variants, which showed an unexpected reduction compared to baseline (Figures S1B and S1D–S1K). A rapid surge of antibody-secreting cells peaked at D+7 and D+9 without altering total B cell ratios (Figures S2A–S2C), indicating targeted activation rather than global expansion and highlighting the importance of early response assessments.

Vaccine-derived, Spike protein subunit 1 (S1)-specific (S1^+^) B cells from blood samples were labeled with a double-conjugated (nucleotide barcode and fluorochrome) S1 bait using the LIBRA-seq technology^37^ and processed using fluorescence-activated cell sorting followed by 5′ single-cell transcriptomics (10× Chromium). This approach enabled us to generate matched scRNA-seq and single-cell BCR sequencing (scBCR-seq) datasets of the same cells, some of which were known to bind the antigen. Guided by the antibody (Figure S1B), cellular (Figure S2B), and repertoire (Figure S3) responses, we selected key time points for this analysis (Figure 1B). In total, we analyzed the transcriptional profile of 27,027 B cells up to week 10 (W10) from four participants, of which 623 were S1^+^. Consistent with serum titers (Figure S1B) and flow cytometry measurements (Figures S2D and S2E), most S1^+^ B cells identified by single-cell transcriptomics were from W10, with smaller numbers observed at D+12 (Figure 1C). A separate single-cell experiment was performed for M6 samples with the aim to evaluate the long-term effects of the vaccine. A total of 7,308 S1^−^ B cells and 674 S1^+^ B cells were identified for this later time point from five participants (Figure 1B). To validate the LIBRA-seq results, we cloned an S1^+^ sequence in three isotype constant regions (IgG1, IgG2, and IgG3) and showed that the three can bind S1 using the ELISA method (Figure S4), supporting the use of LIBRA-seq signals to classify antigen specificity in our single-cell dataset.

Using a previously published transcriptomic atlas of healthy peripheral B cells^16^ we identified 11 B cell clusters or subsets (Figure 1D), each exhibiting distinct transcriptional signatures (Figure S2F; Table S2). When assessed for class-switch status most S1^+^ B cells mapped onto the same uniform manifold approximation and projection (UMAP) space as switched cells (*IGHM−*), memory cells, and the plasmablasts cluster (Figures 1E–1G), indicating the predominant class-switched status of S1^+^ B cells.

Next, we compared the cell type distributions of S1^+^ and S1^−^ B cells (Figures 1H and 1I) and identified statistically significant changes via comparing bootstrapped 95% confidence intervals of cell type proportions, to overcome the sparsity of antigen-specific B cells in this immune response (Figure S5). The antigen-specific (S1^+^) B cell response was characterized by the expansion of classical memory 2 B cells (C_mem2; *SELL*, *CD53*, *ACTB*, *HOPX*, *CRIP1*, *CRIP2*, *S100A10*, *TAGLN2*, *ANXA2*, and *ANXA4*), a rare subtype in S1^−^ B cells, and plasmablasts (plasma; *CD27*, *CD38*, *PRMD1*, *XBP1*, and *JCHAIN*) after primary immunization (D+12) followed by the appearance of S1^+^ double-negative type 2 B cells (DN2; *TBX21*, *ZEB2*, and *FCRL5*) and double-negative type 2 B cells (DN4; *SELL*, *CD53*, *HOPX*, *IGE*, and *IL13RA1*) and a sharp decrease in circulating S1^+^ plasmablasts after the second vaccine dose (W10) (Figures 1H, 1I, and S5). Six months after the initial vaccination (M6), frequencies of C_mem2 and DN2 were reduced, while levels of DN4 S1^+^ B cells surged and became the dominant subset (Figures 1H, 1I, andS5).

Flow cytometry phenotyping corroborated these dynamics, confirming S1^+^ plasmablast (CD27^+^IgD^−^CD24^−^CD38^+^) expansion at D+12 and the increase in S1^+^ DN B cells (CD27^−^IgD^-^), particularly after the second dose (W10) (Figures S6A–S6C). Additionally, the balance between unswitched and switched S1^+^ memory B cells (CD27^+^IgD^−^IgM^+^) shifted toward switched memory B cells (CD27^+^IgD^−^IgM^−^) only after the second dose (W10) (Figures S6B and S6C). In contrast, the phenotypes of S1^−^ B cells remained largely unchanged (Figure 1H), apart from a reduction in both switched and unswitched memory B cells at D+9 and a 2-fold increase in S1^−^ plasmablasts at D+7 (Figure S6D).

These results show that mRNA SARS-CoV-2 vaccination elicited S1^+^ B cells in two waves coinciding with each vaccine dose, inducing switched DN and C_mem2 B cell phenotypes.

### Sterile transcription occurs simultaneously for all *IGHC* genes up to *IGHG2* with certain B cell subsets bypassing this point

**Figure 2:**
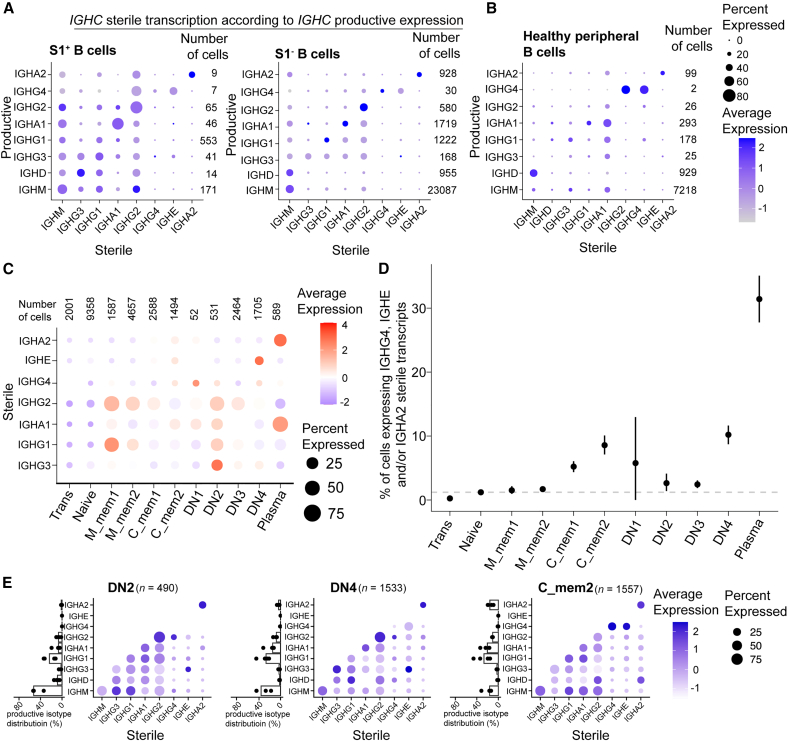
*IGHG2* sterile transcription checkpoint is bypassed by DN and C_mem2 B cells expressing specific productive *IGHC* genes (A and B) Quantification of S1^+^ and S1^−^ B cells in (A) our single-cell (sc) transcriptomic data and (B) a reference dataset of peripheral B cells at homeostasis from Stewart et al.,^16^ in terms of their productive isotype (vertical axis, based on scBCR-seq data) and sterile transcript expression (horizontal axis, quantified using sciCSR). Dot sizes are proportional to amounts of cells positive for the given sterile transcripts, and color depicts expression level. The numbers of B cells expressing each productive transcript are indicated. (C) Quantification of sterile transcription level in each B cell subpopulation in our vaccination scRNA-seq dataset. (D) Proportion of cells in each B cell subpopulation expressing sterile *IGHG4*, *IGHE*, and/or *IGHA2* transcripts *n* = 5. Error bars denote 95% confidence intervals obtained via bootstrapping. The quantification for the naive subpopulation is indicated with a dotted line for ease of comparisons. (E) Quantification separately for the C_mem2, DN2, and DN4 subsets, in terms of their productive BCR isotype distribution (left, bar plot) determined using scBCR-seq data and sterile transcription levels for B cells of different BCR isotypes (right, dot plot). For bar plots, data points correspond to individual donors.

Sterile *IGHC* transcription usually precedes CSR and can be used to predict its likely future directionality.^28^^,^^38^ Hence, we used our recently developed pipeline (sciCSR^18^) to investigate this phenomenon by comparing the levels of productive and sterile *IGHC* transcription in S1^+^ and S1^−^ B cells (Figure 2A). Interestingly, we observed sterile transcription of all *IGHC* genes in the genomic locus up to *IGHG2* in both S1^+^ and S1^−^ B cells (Figure 2A). Exceptions to this arrest included B cells expressing productive transcripts such as *IGHG4* (Figure 2A). To ascertain whether these patterns are specific to the vaccination, analysis of a previously published scRNA-seq data of non-vaccinated healthy control B cells^16^ shows a similar pattern (Figure 2B), confirming that the arrest at *IGHG2* is a general feature of B cells.

Next, we asked whether distinct B cell subsets might exhibit different sterile transcription patterns. We identified DN subsets, both C_mem subsets (especially C_mem2), and plasmablasts as the primary subtypes expressing sterile transcripts beyond the *IGHG2* locus (Figures 2C and 2D). Similar trends were observed when separating S1^+^ and S1^−^ B cells (Figures S7A and S7B), but with larger variability within these subsets in the S1^+^ compartment.

Furthermore, as observed above (Figure 2A), we asked whether sterile transcription was associated with the productive transcription of specific productive *IGHC* at a B cell subset level (Figure 2E). We observed that certain combination of productive and sterile *IGHC* genes predominated in DN2 B cells (i.e., co-expressing of productive *IGHG3* and sterile *IGHE*, or productive *IGHG2* with sterile *IGHG4*), suggestive of non-random class-switch pathways that permit progression beyond the *IGHG2* locus barrier. A similar phenomenon can be observed for DN4 B cells where *IGHE* sterile expression was favored by DN4 B cells expressing productive *IGHG3* and *IGHG4* (Figure 2E). In the case of C_mem2 B cells, sterile *IGHE* was preferentially expressed by C_mem2 B cells expressing productive *IGHG4*. A parallel phenomenon can be observed when separating these B cell subsets by antigen specificity (S1^+^ and S1^−^) (Figure S7C).

This is the evidence that sterile transcription occurs simultaneously for all *IGHC* genes up to *IGHG2*, and that this checkpoint is dependent on a combination of B cell memory subset and productive *IGHC* expression, highlighting that CSR flexibility is B cell subset dependent. This is critical information for B cell-based studies, diagnostics, and immune interventions.

### Class-switch recombination follows a partially sequential pattern up to the *IGHG2* gene locus during SARS-CoV-2 mRNA vaccination

After exploring sterile transcription as a general prelude of CSR during SARS-CoV-2 vaccination, we sought to investigate how this phenomenon might influence CSR at the productive Ig level.

**Figure 3:**
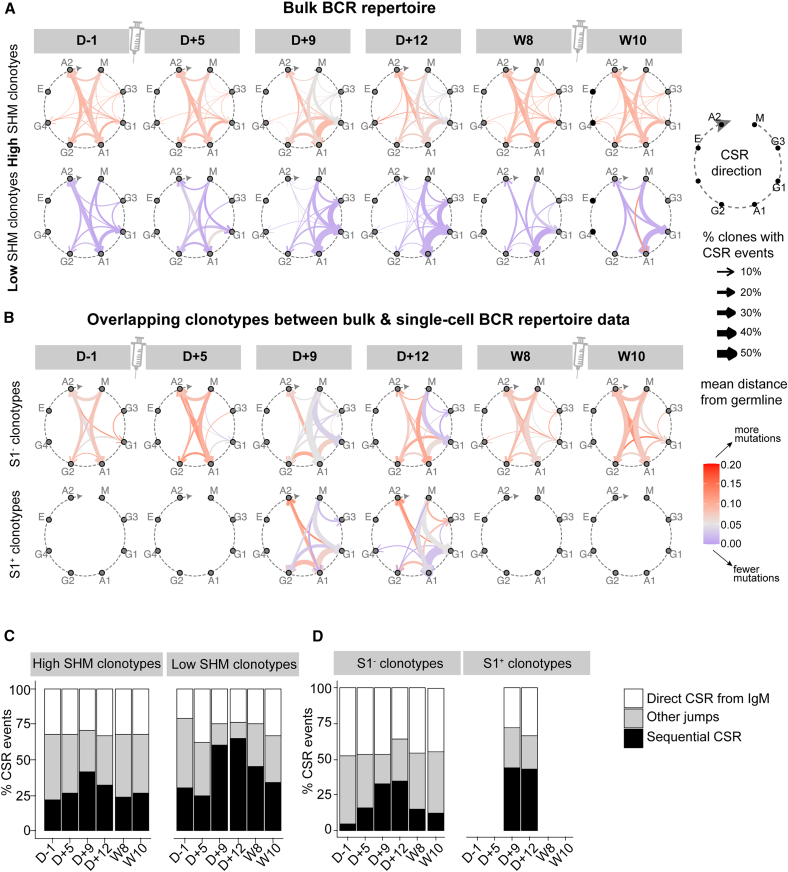
Class-switch recombination in hypomutated, newly vaccine-derived B cells follows a partially sequential pattern up to the *IGHG2* gene locus during SARS-CoV-2 mRNA vaccination (A and B) Longitudinal series of carousel plots showing class switch events of BCR isotypes during the course of vaccination for (A) bulk BCR repertoire (split by High [<99% identity to germline, *n* = 13,401 clonotypes with CSR events detected] or Low [≥99% identity to germline, *n* = 2,018 clonotypes] somatic hypermutation [SHM] levels) and (B) overlapping clonotypes between bulk and single-cell BCR repertoire data (*n* = 357 S1^−^ clonotypes and *n* = 42 S1^+^ clonotypes with CSR events detected). Order of the carrousel matches the physical organization of the arranged clockwise human IGHC gene locus. Arrows connect the start and endpoints of class-switching events, with their width proportional to the frequency of class-switch events and color depicting the mutational level at which class switching occurred. (C and D) Longitudinal quantification of CSR types for Low/High SHM clonotypes (bulk RNA sequencing) (C) and for S1^+^/S1^−^ clones (from single-cell RNA sequencing) (D) according to their nature: direct CSR from IgM, other jumps (start and endpoints of these CSR events are not immediately next to each other physically in the IGH locus), and sequential CSR (the endpoints of these CSR events are directly and immediately 3′ to the starting points).

We leveraged the sequencing depth in the bulk data (a total of 3,778,590 BCR sequences, collapsed into 1,517,840 unique clonotypes) to generate B cell lineage trees, and annotated CSR events across B cell lineages. We observed a shift to lower somatic hypermutation (SHM) levels in the bulk repertoire during the first days of the response, most notably for IgG1 and IgG3 sequences (Figure S3). In addition, the antigen-specific antibody production (Figure S1B) and appearance of S1^+^ B cells (Figure S2E) at these time points suggest that newly derived B cell lineages show low SHM. We, therefore, used low SHM as a proxy for newly derived B cell lineages and analyzed CSR events, defined as direct parent-child relationships in clonotype trees that connect sequences of different isotypes, in Low and High SHM clonotypes separately. Analyzing the directionality of CSR events in these lineages longitudinally, differences between Low and High SHM clonotypes began to emerge as the response peaked, from D+7 and D+9 (Figures 3A, 3C, and S8). Low SHM clonotypes showed a stepwise pattern with *IGHG3* and *IGHG1* switching almost exclusively to *IGHG1* and *IGHA1*, respectively (Figure 3A). Switching events with *IGHM* as origin occurred to *IGHG3* during the peak of the response as well as to *IGHG1* and *IGHA1* (Figure 3A). *IGHA1* was reached via *IGHG1* more often than directly from *IGHM* (Figure 3A). The patterns of switching events toward *IGHA2* were scarce during these time points (Figure 3A). This pattern persisted during the primary response (up to D+19) (Figure S8). Switching beyond *IGHG2* along the locus was limited mainly to *IGHA2* and *IGHG4* to a lesser extent, with very rare *IGHM* to *IGHA2* transitions. Most switching events occurred toward the two genomically adjacent *IGHC*, such as from *IGHM* to *IGHG3* or *IGHG1* and from *IGHG1* to *IGHA1* or *IGHG2* (Figure 3A) in both primary and secondary responses for Low SHM clonotypes. CSR events of Low SHM clonotypes during the secondary response (W10) involved primarily *IGHC* genes downstream of *IGHG3* but exhibited fewer of the stepwise CSR patterns observed earlier in the response (Figure 3A). At W10 there was increased switching toward *IGHA2* from *IGHM*, *IGHG1*, *IGHA1*, and *IGHG2*. We quantified the CSR events in 3 categories: sequential CSR (i.e., the endpoints of these CSR events are directly and immediately 3′ to the starting points), direct CSR from IgM, and other jumps (i.e., start and endpoints of these CSR events are not immediately next to each other physically in the *IGHC* locus) (Figure 3C). This analysis shows an enrichment of sequential CSR by vaccine-derived clones at the peak of the primary response (D+9/D+12) as inferred from the carousel plots (Figure 3A).

In contrast to Low SHM clonotypes, CSR patterns remained largely consistent throughout the time course for High SHM clonotypes, and the stepwise pattern was largely absent (Figures 3A and 3C). Importantly, both Low and High SHM clonotypes indicated a checkpoint at the *IGHG2* level, with switching beyond this point primarily toward *IGHA2* and to a lesser extent to *IGHG4* (Figure 3A), in accordance with the *IGHG2* arrest found in the sterile transcription distribution across the *IGHC* locus (Figure 2).

We next asked whether a similar stepwise CSR pattern can be observed in antigen-specific B cells captured in the single-cell data. To overcome the sparse sampling of S1^+^ B cells, we matched the single-cell and bulk datasets by identifying sequences with identical CDRH3 amino acid sequences (Figure S9A). Using this method, we identified 26,181 S1^−^ and 3,097 S1^+^ sequences with matches across both datasets. Despite only using the heavy-chain CDR3 for matching the single-cell and bulk BCR datasets, we observed substantial CDR3 identity on the light chain (CDRL3) matched within the same clonotype (Figure S9B), suggesting that our approach went beyond coincidental sequence matching and retained heavy-light chain pairing and hence antigen specificity. Importantly, this approach enabled the identification of a greater number of S1^+^-specific sequences for BCR lineage analysis than would be possible by relying solely on scRNA-seq. S1^+^ clonotypes displayed a higher proportion of class-switch event branches in lineage trees compared to S1^−^ clonotypes (Figure S9C) in spite of lower SHM levels in S1^+^ compared to S1^−^ BCR sequences (Figure S9D). SHM levels in S1^+^ B cells increased at M6 compared to D+12 and W10, becoming comparable to the SHM levels in S1^−^ B cells (Figure S9E). Using only these clonotype matches, identifying S1^+^ and S1^−^ cells, we confirmed the CSR preferences shown in the bulk data (Figures 3B and 3D). S1^+^ and S1^−^ clonotypes showed differing patterns of CSR and lower SHM in S1^+^ clonotypes was also noted. These observations gave further support to the stepwise CSR patterns observed in the bulk data (Figures 3A and 3C) being a feature of vaccine-derived antigen-specific B cell lineages.

In summary, we observe that CSR occurs by a combination of direct and stepwise events (a pattern that we term “partially sequential”), with a checkpoint at the *IGHG2* locus. Despite the classical theory of human B cell memory development, linking CSR and SHM, our findings indicate that class-switch recombination in S1^+^ B clonotypes occurs with minimal evidence of SHM in the first 10 weeks of human primary and secondary responses during SARS-CoV-2 mRNA vaccination. These observations go beyond previous experimental settings that had sparse time course coverage and prompt questions as to whether the partially sequential CSR and the *IGHG2* checkpoint are related to the vaccine platform, the antigen, or both. This knowledge has deep implications on how vaccine designs in the future could promote more direct CSR to specific isotypes conducive to immune protection, for example, in requiring extra stimulation to pass the *IGHG2* checkpoint when the mucosal-protective *IGHA2* is required.

### SARS-CoV-2 mRNA vaccine induces prominent IgG1 but limited IgA expression by antigen-specific B cells

Following the observation of differences in the CSR pattern between S1^+^ and S1^−^ clonotypes during SARS-CoV-2 mRNA vaccination and the checkpoint at *IGHG2* (Figure 3), we assessed how this translated into the *IGHC* expression levels and distribution of the different BCR subtypes. This is key as different isotypes play different roles in vaccine protection, efficacy, and transmission prevention, i.e., IgGs prevent systemic and severe disease, while IgAs protect from viral entry and transmission between individuals. Furthermore, subclasses of IgG have variable affinities for the different Fcγ receptors that can alter their physiological function.

**Figure 4:**
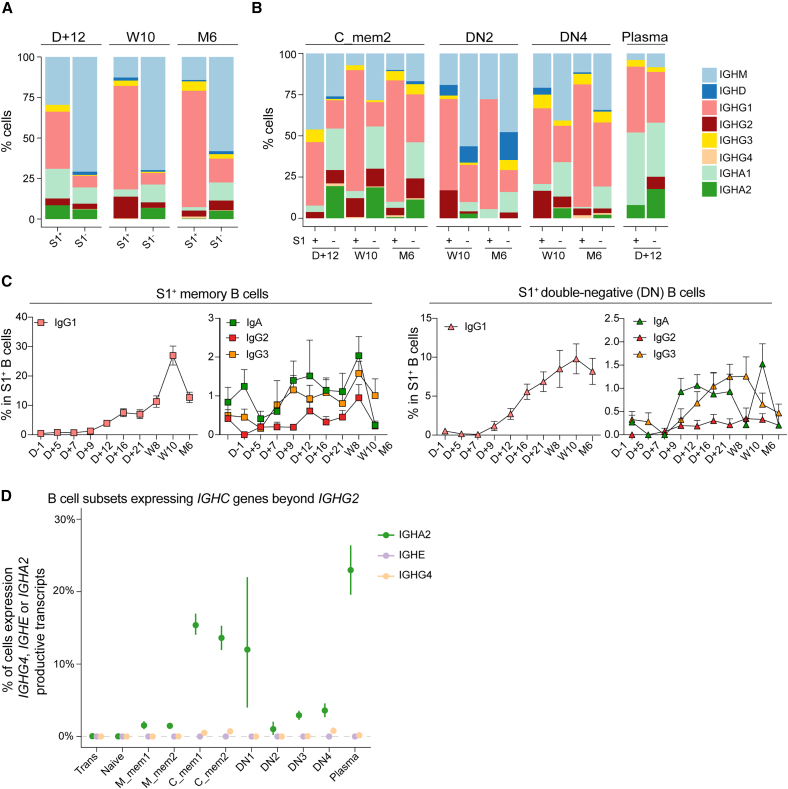
SARS-CoV-2 mRNA vaccine induces prominent IgG1 but limited IgA class switching in S1^+^ B cells (A and B) Comparison of BCR subtype distribution in S1^+^ and S1^−^ B cells at D+12, W10, and M6 time points, over (A) all B cells in the scRNA-seq data and (B) separately quantified within C_mem2, DN2, DN4, and plasmablasts subsets; *n* = 5. Data are only shown if there are more than 10 S1^+^ B cells for the specified time point/cell subset combination. (C) Frequencies of S1^+^ class-switched memory B cells (CD19^+^CD27^+^CD24^+^CD38^lo^IgD^−^IgM^−^, squares) and switched double-negative (DN) B cells (CD19^+^CD27^−^IgD^−^IgM^−^, triangles) as percentage of S1^+^ B cells grouped by BCR isotype quantified using flow cytometry data (from *n* = 12 donors) during vaccine response. Data points and trend lines indicate mean over all donors with available data, and error bars indicate the standard errors of mean. (D) Proportion of cells expressing productive *IGHG4*, *IGHE*, and *IGHA2* (beyond the *IGHG2* checkpoint) in our scRNA-seq dataset grouped by their B cell subpopulations; *n* = 5. Error bars displayed 95% confidence intervals obtained via bootstrapped sampling.

Using scRNA-seq and flow cytometry datasets, we quantified productive *IGHC* transcripts and surface BCR, respectively. Following the first dose (D+12), we observed that although *IGHG1* was the most expressed isotype, *IGHA1* was expressed in a higher percentage of S1^+^ B cells compared to their S1^−^ counterparts (Figure 4A). However, in response to the second dose (W10), *IGHG1* followed by *IGHG2* become the dominant subtypes expressed in S1^+^ B cells, paralleled by a reduction in the use of *IGHA1* (Figure 4A). *IGHG1* remained dominant in S1^+^ B cells by M6 with a reduction of *IGHG2* compared to W10 (Figure 4A). Analysis of productive *IGHC* transcripts by B cell subpopulations showed that S1^+^ C_mem2, DN2, and DN4 cells expressed higher levels of *IGHG1* and much lower levels of *IGHA1* and *IGHA2* compared to their equivalent S1^−^ B cell subsets at all time points (Figure 4B). Notably, the proportion of S1^+^ plasmablasts expressing *IGHA1* at D+12 was very high compared to the proportion of *IGHA1* cells in other S1^+^ B cell subsets (Figures 4B and S10A), indicating that the result of CSR varies according to B cell subset. This is also as inferred by the observation of a differential sterile transcription pattern depending on B cell subset (Figure 2). Changes in the *IGHC* productive expression were validated statistically via comparing bootstrapped 95% confidence intervals of these proportions (Figures S10B and S10C).

Flow cytometry confirmed these findings at the protein level, showing that IgG1^+^ S1^+^ memory (CD27^+^IgD^−^CD24^+^CD38^lo^) and IgG1^+^ DN (CD27^−^IgD^-^) B cell levels remained predominant, especially at W10, while levels of IgA, IgG2, and IgG3 were limited (Figures 4C and S11A–S11C). Additionally, S1^+^ plasmablasts were the main S1^+^ B cell subset expressing IgA at D+12, confirming the scRNA-seq data (Figure S11D). By M6, the frequencies of all switched Ig subtypes within memory B cells, DN B cells, plasmablasts, and total B cells were decreased compared to W10 in S1^+^ B cells, with IgG1 being the main remaining Ig subtype (Figures 4C and S11A–S11E). S1^−^ total, memory, DN B cells, and plasmablasts remained largely unchanged during the study (Figures S11F–S11I).

The dominance of IgG subtypes over IgA subtypes matched with serum antibody titers and BCR bulk repertoire data, showing an expansion of IgG in serum with minimal IgA presence (Figure S1B) and an increase in the IgG1 compartment in the bulk repertoire following the initial vaccination (Figures S3C–S3D).

Finally, we hypothesized that the checkpoint at *IGHG2* observed in all B cells at the level of sterile transcription (Figure 2) and CSR (Figure 3) could influence the expression of productive *IGHG4*, *IGHE*, and *IGHA2* (i.e., the *IGHC* genes beyond *IGHG2* in the genomic locus) and be dependent on B cell subset. Indeed, as observed when analyzing the sterile transcription by subset (Figure 2), we observed that expression of productive transcripts beyond the *IGHG2* was possible but varied greatly according to the B cell subset (Figure 4D). Furthermore, the main productive *IGHC* expressed beyond *IGHG2* was *IGHA2*, corroborating the transcriptomic data on CSR events (Figure 3).

These data show that, despite observing sequential switching to isotypes downstream of *IGHG1* (i.e., *IGHA1*), most of the vaccine-derived antigen-specific B cells were *IGHG1* during the primary response, apart from plasmablasts. Indeed, high IgG1 expression has been observed by others during COVID-19^39^^,^^40^ and SARS-CoV-2 vaccination^41^ as well as during other viral infections such as Ebola, influenza, and respiratory syncytial virus.^8^^,^^42^^,^^43^^,^^44^^,^^45^^,^^46^ Furthermore, expression of sterile transcripts beyond *IGHG2* according to the B cell maturation subset correlates with their expression of productive transcripts. This knowledge is key in understanding how vaccination induces specific antibody isotypes and which memory B cells can express them, therefore enabling design of therapeutic interventions by adjusting *IGHC* expression.

### VDJ gene usage is dependent on BCR isotype and is associated with CSR temporal variation during SARS-CoV-2 vaccination

A successful B cell response requires both CSR and the optimization of antigen specificity governed by variable (V), diversity (D), and joining (J) segment usage. Despite both processes being crucial for the function of the resulting antibody/BCR, little is known about the interaction between these in the context of human primary response. This is key knowledge as VDJ gene usage determines antigen specificity during vaccination, allergy, and autoimmunity.

**Figure 5:**
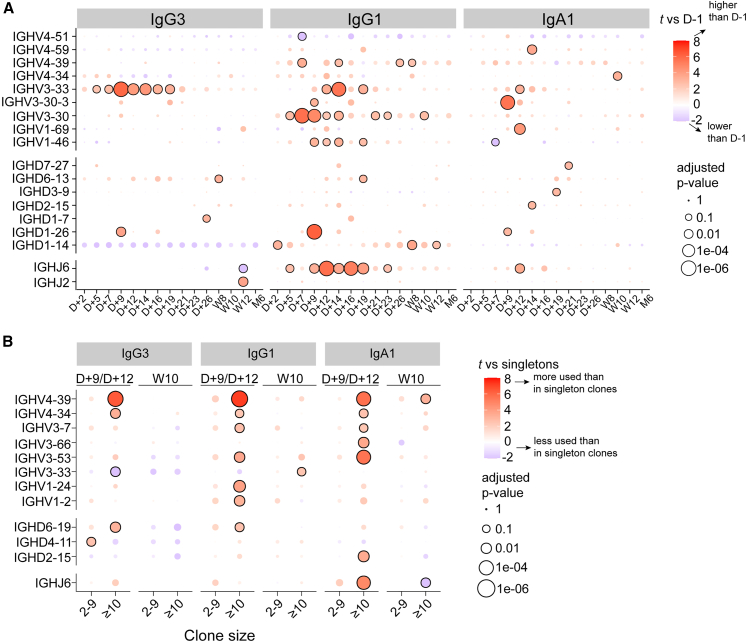
VDJ gene usage is dependent on BCR isotype and transiently polyclonal (A) Change in immunoglobulin heavy-chain variable (V), diversity (D), and J (joining) gene usage across time points and BCR isotypes in the bulk BCR sequencing data. Gene usage was evaluated on subsets of BCR sequences of different isotypes: IgG3 (*n* = 30,451), IgG1 (*n* = 300,757), and IgA1 (*n* = 407,439). Statistical significance was assessed by fitting mixed-effect linear models of percentage gene usage (dependent variable) against time point as the fixed effect and donor identifiers as the random effect. Bubble color depicts effect size compared to D−1 (positive value indicates elevated usage of gene compared to D−1), and bubble sizes correspond to *p* value after false discovery rate adjustment. (B) Comparison of heavy-chain V, D, and J gene usage as shown in (A) between clonotypes of different sizes. Gene usage was computed for sequence subsets as defined in (A), separately for time points at the first peak (D+9 and D+12) and the second peak (Wk10). Clonotypes were grouped by their sizes, into singletons, clonotypes with 2–9 sequences, and those with more than 10 sequences (“≥10”). Mixed effect models were fitted to compare gene usage against singleton clonotypes as a control, with clonotype sizes as fixed effects and donors as random effects.

We analyzed VDJ usage relative to the baseline (D−1) of those isotypes that increase during the primary response (Figures S3C and S3D) and observed that certain isotypes were preferred by specific *IGHV* genes (Figure 5A). For instance, some genes such as *IGHV3-33* and *IGHD1-26* are used by IgG3, IgG1, and IgA1, while usage of others such as *IGHV4-59*, *IGHV3-30-3*, and *IGHV1-69* was specifically elevated for IgA1 (Figure 5A). In addition to the association of VDJ genes with BCR isotype, VDJ gene usage varied according to the time point with some genes appearing earlier (*IGHV3-30* and *IGHV3-33*), and others appearing later (*IGHV4-59* and *IGHD2-15*) during the primary response (Figure 5A). Furthermore, some *IGHV* genes were more predominant in larger clonal expansions (Figure 5B). Among others, the *IGHV3-53* gene, frequently associated with SARS-CoV-2-neutralizing antibodies,^47^ was notably enriched in larger clonotypes.

Motivated by the variability in the temporal evolution of different *IGHV* gene usage (Figure 5A), we next investigated the relation of these genes with CSR in a temporal manner focusing on CSR events with low SHM as a proxy for vaccine-specific clones (Figure 3A, S9F, and S9G). We found that clonotypes using certain V genes (i.e., *IGHV3-30-3*, Figure S12A) started CSR as early as D+7, while others such as *IGHV3-30* underwent CSR during a shorter time frame, between D+12 and D+16 (Figure S12B). Finally, a third pattern was observed (i.e., *IGHV1-69*, Figure S12C) where the start of CSR response was delayed but persisted until D+19. This highlights variations in CSR timing and trajectories associated with specific V gene usage. Here, we also observed that the CSR *IGHG2* checkpoint was present in all analyzed V genes, with occasional differences between clones utilizing different V genes, e.g., a stronger switching toward *IGHG4* in the *IGHV1-69* gene in comparison to *IGHV3-30* and *IGHV3-30-3* genes (Figures S12A–S12C).

When the same data are analyzed regardless of the isotype, a transient increased representation of several V, D, and J genes (e.g., *IGHV3-33*, *IGHV3-30*, *IGHV*3-13, *IGHD1-26*, and *IGHJ6*) is observed during the first peak (D+9/+12), while only the *IGHV4-34* gene, previously found to be overexpressed in hospitalized COVID-19 patients,^8^ was upregulated during secondary response peaks (W10) (Figure S13), suggesting a polyclonal primary response but an oligoclonal response after the second dose.

Complementarity-determining region 3 (CDRH3) of *IGH* genes encodes a crucial antigen-binding region of the antibody and can often be seen to be altered at the population level in response to immune challenge. Bulk BCR sequencing data showed increased length and hydrophobicity in the CDRH3 regions of *IGHG1* sequences at D+12 (Figures S14A and S14B), which aligned with previous findings,^8^ and correlated with RBD-specific IgG titers (Figure S14C). We corroborated CDRH3 features with our data on antigen-positive cells from scBCR-seq. BCR *IGHV* sequences of S1^+^ also had significantly longer and more hydrophobic CDRH3 regions than S1^−^ B cells (Figures S14D and S14E). When compared by *IGHC* subtype, S1^+^ expressing *IGHG1* and *IGHG2* exhibited longer CDRH3 regions than their S1^−^ B cells (Figure S14F). Similarly, CDRH3 regions in S1^+^ B cells that express the *IGHG1*, *IGHG2*, *IGHM*, and *IGHA1* subtypes are more hydrophobic compared to the same CDRH3 regions in S1^−^ B cells (Figure S14G).

Collectively, these results show that VDJ usage is dependent on several factors such as BCR isotype, response phase (primary vs. secondary), clone size, and that it influences the timing of CSR. These are crucial pieces of knowledge to inform how to best design vaccines with a desired functional aim as VDJ usage determines antigen specificity. Furthermore, the same principle could be applied to antibody-based diseases such as autoimmunity or allergy where the antigen and hence the VDJ usage is pivotal. This could mean that certain auto-antigens or allergens might favor certain CSR patterns, which in turn results in specific isotypes having a higher pathogenic potential.

## Discussion

Our study provides a multi-immunomic resource for studying the dynamics of the human primary immune response. The intensive sampling schedule, particularly within the first 3 weeks of the initial vaccine dose, has enabled unparalleled temporal resolution of early B cell subset dynamics, BCR repertoire, VDJ gene usage, and CSR. This approach provides insights into the evolution of the immune response, offering a deeper understanding of B cell development compared to previous studies.^48^^,^^49^^,^^50^^,^^51^^,^^52^^,^^53^^,^^54^^,^^55^^,^^56^^,^^57^ We have used these data to finely map the temporal evolution of the B cell response, integrating the data from serology with those from flow cytometry, bulk BCR repertoire, scRNA-seq, and scBCR-seq to compare critical events and peaks of responses between observational methods and revealing crucial insights into the regulation of antibody CSR.

The central discovery in these data are the openness of the *IGHC* locus to enable sequential switching, but mainly up to the *IGHG2* gene. This was apparent not only in the productive transcripts of B cells but also in the patterns of sterile transcription in the scRNA-seq data. Thus, it appears that activation of B cells causes sterile transcription of all *IGHC* genes as far as *IGHG2*, and only in certain circumstances will transcripts occur past this point. These circumstances involve multiple B cell developmental changes since we find that the exceptions to the *IGHG2* arrest (in both S1^+^ and S1^−^ B cells) were in particular types of B cell, namely, the DN and C-mem2 subsets. Moreover, we found that certain B cell subsets expressing specific BCR isotypes expressed sterile transcripts beyond *IGHG2* (i.e., DN2 and DN4 with productive IgG3 expressing sterile *IGHE*). Additionally, stimuli such as cytokines and pathogen are also likely to influence the sterile transcription beyond this point. Indeed, such a constraining mechanism may have evolved to prevent inappropriate class switching to IgE and to limit Th2-driven antibody responses, which are typically restricted to specific immune contexts, e.g., in the defense against helminth infections. Similarly, the lack of switching to IgE and IgG4 could be due to the tight regulation of IgE-B cells by several mechanisms^58^^,^^59^ and the specific circumstances in which a non-inflammatory isotype such as IgG4 is needed.^60^ The fact that all sterile transcripts up to *IGHG2* are present in newly activated B cells also raises the important question of CSR control in a negative fashion. For instance, if the intention is to promote CSR to IgG1, then instead of looking for mechanisms targeted toward *IGHG1*, our results highlight the need to discover mechanisms positively targeting *IGHG1*. Our results highlight the need to discover mechanisms that prevent CSR beyond *IGHG1*, such as avoiding positive signals allowing continuation of CSR or identifying potential inhibitors. This study describes these phenomena in great detail, and these results are key for understanding the immune response and CSR dynamics in naive individuals.

Organization of the BCR data into lineages enables the study of CSR events as they occur along the timeline. Integrating the scBCR-seq with bulk data overcomes the limitations that occur with the data types individually, allowing exploration of specificity as well as the volume of data needed to provide sufficient lineages with a minimum risk of identifying non-antigen-specific heavy chains in the bulk dataset. The steady-state picture at D−1 is similar to that published by Horns et al.,^34^ acknowledging that switching can occur both directly from IgM to another subclass and between subclasses. However, a static picture fails to capture intermediate stages, and these data enable a distinction between background steady state and antigen labeled (by scRNA-seq) or likely antigen specific (as inferred by appearance of hypomutated sequences) over a detailed time series. The refined picture that emerges is of a “partial sequential” switching, with some direct routes from IgM and some routes that proceed via closer downstream *IGHC* genes as the immune response proceeds. The switching begins from upstream isotypes such as IgM, IgG3, and IgG1 during a primary response, but secondary responses had downstream originators such as IgG1 and IgA1. This might be indicative of a mature CSR response compared to the CSR occurring during the primary response where memory B cells, without germinal center experience, undergo subsequent rounds of switching toward isotypes further down the genome. The fact that some early switching events start from IgG3 and IgA1 suggests a possible pre-existing memory due to cross-reactivity.^61^^,^^62^^,^^63^ Switching to IgA2 mostly arises via an IgA1 intermediate. This information is crucial for researchers who aim to elicit IgA2 responses, for example, in mucosal vaccination.

SHM and CSR have been tightly linked as both require the activation-induced cytidine deaminase enzyme, thought to be restricted to the germinal center.^64^ However, there is a disconnect between these two processes as we observe CSR long before accumulation of typical levels of SHM.^38^ Classically, from studies in mice, it has been thought that primary T cell-dependent immune responses showed early accumulation of hypermutation (around 7 days post-immunization).^65^^,^^66^ However, recent evidence in mice shows that CSR occurs at the follicle edge and is decoupled from SHM^38^ and that IgG-switched memory B cells show low or no mutation, which support our observations.^67^^,^^68^ Here, we found that hypomutation persisted throughout most of our sampling time course in S1^+^ B cells and clones, even at the peak of the secondary response (W10), an observation not previously reported in mice or humans after secondary immunizations, especially with such a temporal delay. Hypomutation of switched S1^+^ B cells has also been previously reported in COVID-19 disease.^69^ One possible explanation is that S1^+^ B cells do not need SHM. However, this is not likely the case since by month 6 the SHM levels are similar to the rest of memory B cells. Supporting our evidence, recent observations of fine-needle aspirate samples from human lymph nodes also show a slow and prolonged affinity maturation in the human germinal center.^70^

Antibody class switching without SHM could suggest an extrafollicular response by S1^+^ B cells in the early stages as B cells that have not entered the germinal center usually show low mutation rates.^71^^,^^72^^,^^73^ The nature of the vaccine, containing mRNA, could be relevant here since RNA can trigger a type 1 interferon reaction known to favor extrafollicular responses.^74^ The expansion of DN2 B cells would concur with this possibility.^75^ Indeed, several studies report extrafollicular responses and increased DN during COVID-19 and SARS-CoV-2 vaccination.^76^^,^^77^^,^^78^^,^^79^ Antigen-specific DN2 was greatly downregulated in the long term, while mutation rates increased in S1^+^ B cells, which could be attributed to the development of canonical follicular and GC responses.

In addition to CSR showing specific patterns and experiencing a checkpoint after *IGHG2*, we find that this process can also be influenced by the VDJ gene usage. We show that certain isotypes are preferred in combination with specific sets of VDJ genes. Moreover, the *IGHV* gene usage also seems to be associated with the temporal occurrence of CSR with some *IGHV* genes undergoing CSR early during the response, while others undergo delayed CSR. Furthermore, despite CSR patterns being similar for all *IGHV* genes, we observed that some *IGHV* genes experience differential *IGHG2* checkpoint bypassing in comparison to others. Hence, VDJ gene usage and constant region isotype should be seen as co-dependent features of antibody/BCR, as CSR can be influenced by the VDJ gene usage. This is important knowledge to inform how to best design vaccines with a desired functional aim through specific isotypes, as VDJ usage determines antigen specificity. In addition, specific VDJ usage in antibody-based diseases such as autoimmunity or allergy could influence a more pathogenic CSR.

In summary, we present a multi-omic dataset that can serve as a blueprint to analyze CSR and other aspects of B cell response during primary human immune challenge. Our observations complement the way we think about the dynamics of CSR, showing generalized activation of CSR up to *IGHG2* with B cell development and Ig-subtype-specific states allowing a breach of the checkpoint at *IGHG2*. We propose that this checkpoint acts as a natural barrier, limiting excessive switching to downstream isotypes with potentially adverse effects such as IgE and finely tuning the production of mucosal pathogen clearing IgA2 and non-inflammatory isotypes IgG4. Indeed, *IGHA2* is mostly achieved via *IGHA1*, and a failure to stop CSR at *IGHG1* will likely result in accumulation of IgG2 instead of IgG1. These are critical considerations to be made in the design of immune interventions such as vaccines or immunomodulatory therapies.

This research could be used as a cornerstone of a generalizable model of CSR by comparative analysis against other vaccination and infection studies in naive individuals in the future. Our scRNA-seq data can serve as an atlas of B cell states for discovering new genes involved in the regulation of CSR and other aspects of B cell maturation, and as a general resource to interrogate proven CSR-regulating molecules and pathways identified in animal models. Identifying similarities and differences in clonal dynamics, analyzing how CSR processes intersect with BCR clonal diversification and affinity maturation, together with integrating these findings with experimental or computational assessments of antigen specificity in different settings will advance our knowledge of human B cell activation. This is required to improve vaccines that can cause switching beyond IgG to favor IgA1 and IgA2 for greater mucosal protection,^80^^,^^81^^,^^82^ or to be applied to better understand and optimize CSR in antibody-driven diseases such as for autoimmunity and allergy.

### Limitations of the study

A limitation of our methodology is the sampling bias, particularly in single-cell transcriptomics, where we cannot sample all existing B cells and clones. This means that we may have missed intermediate steps of some of the direct switching events we observed, overestimating the proportion of direct switches. Similarly, in order to maximize the chances of seeing class-switched lineages rather than naive B cell data, we sequenced equivalent amounts of IgM, IgG, and IgA, which may have caused an underestimation of the proportion of direct switches. Sampling bias is an important aspect to note in all studies involving BCR repertoire with our data highlighting the limited overlap one can get in matching samples from the same person; even in the larger S1^−^ scBCR-seq data, there was a low overlap of clonotypes between the single-cell and bulk datasets. In addition, we sampled peripheral immune cells without assessing tissue residency. Consequently, the lower presence of IgA^+^ S1^+^ B cells could be due to a migration of these to mucosal surfaces. However, the evidence does not support this as studies have found that SARS-CoV-2 infection, but not intramuscular vaccination, elicits IgA^+^ B cell residency.^83^ Indeed, we corroborated this as we observed IgA secretion in the saliva of COVID-19 patients but not in vaccinees. Detecting antigen-specific IgG plasmablasts is difficult due to their lower surface BCR expression compared with IgA-expressing cells.^84^^,^^85^ As a result, some S1-specific IgG plasmablasts may have been missed by flow cytometry and scRNA-seq, which could have overrepresented the presence of IgA plasmablasts. Finally, some features of the observed immune response may be attributable exclusively to the mRNA vaccine platform such as the increase of DN B cells and BCR hypomutation, as other human studies, using protein-,^86^^,^^87^ vector-based,^88^^,^^89^^,^^90^^,^^91^^,^^92^^,^^93^ or inactivated vaccine platforms,^94^^,^^95^ have not investigated or found the presence of DN or atypical memory B cells in humans.

## Resource availability

### Lead contact

Further information and requests for resources and reagents should be directed to and will be fulfilled by Deborah K. Dunn-Walters (d.dunn-walters@surrey.ac.uk) upon request.

### Materials availability

This study did not generate new unique reagents.

### Data and code availability

• The multi-omics data generated in this study can be viewed and queried on a dedicated web-based viewer at https://fraternalilab.cs.ucl.ac.uk/CovVaxBcells/. Raw single-cell sequencing data are deposited at ArrayExpress with accession E-MTAB-16531. Bulk BCR sequencing data are available via Zenodo at https://doi.org/10.5281/zenodo.18187851.
• This paper does not report original code.
• Any additional information required to reanalyze the data reported in this paper is available from the lead contact upon request.

## Acknowledgments

The authors thank the participants of the study for their time and willingness to donate samples during the thorough schedule. BioRender.com was used to draw the graphical abstract and the schematic illustrating sample collection timeline in Figure 1A. The authors thank the members of the Research Facility at the Institute of Immunity and Transplantation (University College London). This work was funded by the 10.13039/501100000268Biotechnology and Biological Sciences Research Council (BB/T002212/1 with F.F. as principal investigator). E.S. was funded by a personal PhD fellowship by the 10.13039/501100003513University of Surrey. The funders had no role in the collection and analysis of the samples, in the interpretation of data, in writing the report, or in the decision to submit the paper for publication.

## Author contributions

G.M.-G., A.T.S., P.B., D. Kateregga, E.S., C.J.M.P., and Z.B. performed sample collection and processing. A.G., D.K.D.-W., and C.M. planned sample collection. G.M.-G., J.C.F.N., A.T.S., P.B., F.F., D.K.D.-W., and C.M. designed the experiments. G.M.-G., A.T.S., E.S., B.B.M., and Y.H.G. performed the experiments. G.M.-G., J.C.F.N., A.T.S., E.S., D.K.D.-W., D. Kipling, D.G., and L.S. analyzed data. P.B., F.F., D.K.D.-W. and C.M. provided supervision for experimental design and data analysis. G.M.-G., J.C.F.N., A.T.S., F.F., D.K.D.-W., and C.M. prepared the manuscript. All authors read, commented, and approved the final manuscript.

## Declaration of interests

The authors declare no competing interests.

## STAR★Methods

### Key resources table

**Table:** 

REAGENT or RESOURCE	SOURCE	IDENTIFIER
**Antibodies**		

AF700-CD45 (clone 2D1); dilution 1:50 (whole blood for analyzer)	BioLegend	Cat# 368513; RRID: AB_2566373
PE/Dazzle™ 594-CD20 (clone 2H7); dilution 1:20 (whole blood for analyzer)	BioLegend	Cat# 302347; RRID: AB_2564386
APC-Cy7-IgM (clone MHM-88); dilution 1:10 (whole blood for analyzer)	BioLegend	Cat# 314519; RRID: AB_10897095
BV510-CD138 (clone MI15); dilution 1:20 (whole blood for analyzer)	BioLegend	Cat# 356517; RRID: AB_2562661
BUV737-HLA-DR (clone Tu39); dilution 1:20 (whole blood for analyzer)	BD Biosciences	Cat# 741845; RRID: AB_2871179
BV421-CD21 (clone B-ly4); dilution 1:20 (whole blood for analyzer)	BD Biosciences	Cat# 562966; RRID: AB_2737921
PE-Cy5-CD3 (clone HIT3a); dilution 1:25 (whole blood for analyzer)	BioLegend	Cat# 300310; RRID: AB_314046
PE-Cy5-CD14 (clone M5E2); dilution 1:25 (whole blood for analyzer)	BioLegend	Cat# 301864; RRID: AB_2860767
BV605-CD38 (clone HB-7); dilution 1:20 (whole blood for analyzer) or 1:200 (PBMCs for analyzer)	BioLegend	Cat# 356642; RRID: AB_2820009
PE-Cy7-CD24 (clone ML5); dilution 1:20 (whole blood for analyzer) or 1:200 (PBMCs for analyzer)	BioLegend	Cat# 311120; RRID: AB_2259843
BV785-CD19 (clone HIB19); dilution 1:20 (whole blood for analyzer) or 1:200 (PBMCs for analyzer)	BioLegend	Cat# 302240; RRID: AB_2563442
BUV395-CD27 (clone M-T271); dilution 1:20 (whole blood for analyzer) or 1:200 (PBMCs for analyzer)	BD Biosciences	Cat# 740291; RRID: AB_2740030
PerCP-Cy5.5-IgD (clone IA6-2); dilution 1:10 (whole blood for analyzer) or 1:200 (PBMCs for analyzer)	BD Biosciences	Cat# 561315; RRID: AB_10646033
APC-Fire 750-IgM (clone MHM-88); dilution 1:200 (PBMCs for analyzer)	BioLegend	Cat# 314546; RRID: AB_2800834
PE-IgG1 (clone SAG1); dilution 1:400 (PBMCs for analyzer)	Cytogonos	Cat# CYT-IGG1PE; RRID: AB_3674600
PE-IgG2 (clone SAG2); dilution 1:400 (PBMCs for analyzer)	Cytogonos	Cat# CYT-IGG2PE; RRID: AB_3720081
FITC-IgG2 (clone SAG2); dilution 1:400 (PBMCs for analyzer)	Cytogonos	Cat# CYT-IGG2F; RRID: AB_3720082
FITC-IgG3 (clone SAG3); dilution 1:400 (PBMCs for analyzer)	Cytogonos	Cat# CYT-IGG3F; RRID: AB_3720871
PE-Vio615-IgA (clone REA1014; dilution 1:200 (PBMCs for analyzer)	Miltenyi	Cat# 130-116-882; RRID: AB_2727740
Spark-NIR 685-CD3 (clone SK7); dilution 1:200 (PBMCs for analyzer)	BioLegend	Cat# 344862; RRID: AB_2860899
Spark-NIR 685-CD14 (clone S18004B); dilution 1:400 (PBMCs for analyzer)	BioLegend	Cat# 399209; RRID: AB_2894513
BUV563-CD21 (clone B-ly4); dilution 1:200 (PBMCs for analyzer)	BD Biosciences	Cat# 741362; RRID: AB_2870862
BV650-CD71 (clone CY1G4); dilution 1:200 (PBMCs for analyzer)	BioLegend	Cat# 334116; RRID: AB_2687103
BV480-CD11c (clone B-ly6); dilution 1:200 (PBMCs for analyzer)	BD Biosciences	Cat# 566135; RRID: AB_2739534
BV750-CD73 (clone AD2); dilution 1:400 (PBMCs for analyzer)	BD Biosciences	Cat# 747205; RRID: AB_2871931
BUV496-Fcrl4 (clone A1); dilution 1:200 (PBMCs for analyzer)	BD Biosciences	Cat# 750568; RRID: AB_2874703
PE-Fire 810-CD39 (clone A1); dilution 1:100 (PBMCs for analyzer)	BioLegend	Cat# 328245; RRID: AB_2894563
APC-Fire 810-CD95 (clone DX2); dilution 1:200 (PBMCs for analyzer)	BioLegend	Cat# 305663; RRID: AB_2894542
BV570-CD20 (clone 2H7); dilution 1:100 (PBMCs for analyzer)	BioLegend	Cat# 302332; RRID: AB_2563805
AF700-CD45R (clone MEM-55); dilution 1:100 (PBMCs for analyzer)	exbio	Cat# A7-224-T100; RRID: AB_10733799
BUV661-CD126 (clone M5); dilution 1:100 (PBMCs for analyzer)	BD Biosciences	Cat# 752527; RRID: AB_2917517
BUV615-CD268 (clone 11C1); dilution 1:200 (PBMCs for analyzer)	BD Biosciences	Cat# 751241; RRID: AB_2875261
BV711-CD267 (clone 1A1-K21-M22); dilution 1:200 (PBMCs for analyzer)	BD Biosciences	Cat# 744147; RRID: AB_2742033
PE-Fire 700-CD185 (clone J252D4); dilution 1:100 (PBMCs for analyzer)	BioLegend	Cat# 356954; RRID: AB_2894489
BUV805-CD183 (clone 1C6/CXCR3); dilution 1:100 (PBMCs for analyzer)	BD Biosciences	Cat# 742048; RRID: AB_2871338
PerCP-eFluor 710-CD360 (clone 2 S × 21 R); dilution 1:100 (PBMCs for analyzer)	eBioscience	Cat# 46-3601-42; RRID: AB_2573751
BV421-CD19 (clone HIB19); dilution 1:200 (PBMCs for sorting)	BioLegend	Cat# 302234; RRID: AB_11142678
BV785-CD14 (clone 63D3); dilution 1:200 (PBMCs for sorting)	BioLegend	Cat# 367141; RRID: AB_2810578
TotalSeq anti-B2M hashtag 1 (clone LNH-94, 2M2)	BioLegend	Cat# 394661; RRID: AB_2801031
TotalSeq anti-B2M hashtag 2 (clone LNH-94, 2M2)	BioLegend	Cat# 394663; RRID: AB_2801032
TotalSeq anti-B2M hashtag 3 (clone LNH-94, 2M2)	BioLegend	Cat# 394665; RRID: AB_2801033
TotalSeq anti-B2M hashtag 4 (clone LNH-94, 2M2)	BioLegend	Cat# 394667; RRID: AB_2801034
TotalSeq anti-B2M hashtag 5 (clone LNH-94, 2M2)	BioLegend	Cat# 394669; RRID: AB_2801035
TotalSeq anti-B2M hashtag 6 (clone LNH-94, 2M2)	BioLegend	Cat# 394671; RRID: AB_2820042
Positive anti-S1 IgM control ELISA (clone CR3022)	Absolute Biotech	Cat# Ab01680-15-0; RRID: AB_3720872
Positive anti-S1 IgA control ELISA (clone CR3022)	Absolute Biotech	Cat# Ab01680-16-0; RRID: AB_3720873
Positive anti-S1 IgG control ELISA (clone CR3022)	Abcam	Cat# ab273073; RRID: AB_3073570
HRP-conjugated anti-human IgM (polyclonal)	ThermoFisher	Cat# A18835; RRID: AB_2535612
HRP-conjugated anti-human IgA (polyclonal)	Sigma-Aldrich/Merk	Cat# A0295-1ML; RRID: AB_257876
HRP-conjugated anti-human IgG (polyclonal)	ThermoFisher	Cat# A18817; RRID: AB_2535594
Biotin-conjugated goat anti-human IgG (polyclonal)	Sigma-Aldrich/Merk	Cat# AP112B; RRID: RRID:AB_92429

**Biological samples**		

PBMCs	This study	N/A
Serum	This study	N/A
Saliva	This study	N/A
Saliva from SARS-CoV-2 infected individuals	Stewart, Sinclair, Ng et al.^8^	N/A

**Chemicals, peptides, and recombinant proteins**		

Streptavidin-BV421 1 (S1)	Biolegend	Cat# 405225
Streptavidin-APC 2 (S1)	Biolegend	Cat# 405207
Streptavidin-BUV737 (RBD)	BD Biosciences	Cat# 612775
Streptavidin-PE-Cy5 (Decoy, *ex vivo* B cell phenotyping)	Biolegend	Cat# 405205
Streptavidin-FITC (Decoy, sorting)	Biolegend	Cat# 405201
D-biotin	SigmaAldrich	Cat# B-4501
TotalSeq™ streptavidin-PE	Biolegend	Cat# 405261
TotalSeq™ streptavidin-APC	Biolegend	Cat# 405283
TotalSeq™-C0971 Streptavidin	Biolegend	Cat# 405271
Biotin-S1	Biolegend	Cat# 793806
Biotin-RBD	Biolegend	Cat# 793904
RBD for ELISA binding assay	Abcam	Cat# ab273065
S1 for ELISA binding assay	R&D Systems	Cat# 11058-CV
FcR blocking agent	Miltenyi	Cat# 130-059-901

**Critical commercial assays**		

V-PLEX SARS-CoV-2 panel 30 (ACE2)	Meso Scale Diagnostics (MSD)	Cat# K15635U
Chromium Next GEM Single cell 5′ reagent kit v2 (Dual Index)	10x	Cat# PN-1000265

**Deposited data**		

Single-cell transcriptomics and BCR sequencing data generated in this manuscript	This manuscript	ArrayExpress: E-MTAB-16531
Bulk BCR repertoire data generated in this manuscript	This manuscript	https://doi.org/10.5281/zenodo.18187851
Whole blood flow cytometry data generated in this manuscript	This manuscript	https://fraternalilab.cs.ucl.ac.uk/CovVaxBcells/
Antigen-specific B cell flow cytometry data generated in this manuscript	This manuscript	https://fraternalilab.cs.ucl.ac.uk/CovVaxBcells/
Antibody titer measurement generated in this manuscript	This manuscript	https://fraternalilab.cs.ucl.ac.uk/CovVaxBcells/
Peripheral blood B cell single-cell transcriptomics dataset from healthy individuals	Stewart et al.^16^	ArrayExpress: E-MTAB-9544

**Software and algorithms**		

GraphPad Prism (v9)	Domatics	https://www.graphpad.com
FlowJo (v10.8.1)	BD Biosciences	https://www.flowjo.com
cellranger (v6.1.2)	10x Genomics	https://www.10xgenomics.com/support/software/cell-ranger/
phylip (v3.695)	Felsenstein^92^	https://phylipweb.github.io/phylip/
IgBLAST (v1.19.0)	National Center for Biotechnology Information (NCBI)	https://ncbi.github.io/igblast/
IMGT/HighV-Quest web-server	Alamyar et al.^96^	https://www.imgt.org/HighV-QUEST/
BRepertoire web-server	Margreitter et al.^97^	https://brepertoire.cs.ucl.ac.uk/
TotalVI from scvi-tools (v0.20.0)	Gayoso et al.^98^	https://github.com/scverse/scvi-tools
R v4.2.0	Comprehensive R Archive Network (CRAN)	https://cran.r-project.org/
Peptides v2.4.4	CRAN	https://cran.r-project.org/web/packages/Peptides/index.html
BrepPhylo v0.4.2	Stewart et al.^8^	https://github.com/Fraternalilab/BrepPhylo
Seurat v4.3.0	Hao et al.^99^	https://cran.r-project.org/web/packages/Seurat/index.html
sciCSR v0.3.3	Ng et al.^18^	https://github.com/Fraternalilab/sciCSR
TIgGER v1.1.0	Gadala-Maria et al.^100^	https://cran.r-project.org/web/packages/tigger/index.html
ggplot2 v3.4.1	CRAN	https://cran.r-project.org/web/packages/ggplot2/index.html
boot v1.3-28	CRAN	https://cran.r-project.org/web/packages/boot/index.html
lmerTest v3.1-3	CRAN	https://cran.r-project.org/web/packages/lmerTest/index.html

**Other**		

LSR II Flow Cytometer	BD Biosciences	https://www.bdbiosciences.com/en-gb/products/instruments/flow-cytometers
FACSAria™ Fusion Flow Cytometer (sorter)	BD Biosciences	https://www.bdbiosciences.com/en-gb/products/instruments/flow-cytometers
Cytek Aurora 5 Lasers	Cytek	https://cytekbio.com/blogs/resources/fluorochrome-guide-5l-uv-v-b-yg-r
SpectraMax iD3 plate reader	Molecular Devices	https://www.moleculardevices.com
10X chromium controller	10x	https://www.10xgenomics.com/instruments/chromium-controller

### Experimental model and study participant details

#### Study design and participants

This study was designed to investigate the immune response to the SARS-CoV-2 vaccine with particular focus on CSR and B cell responses in a highly systematic timeline to analyze immune processes in a dynamic way. Fifteen healthy adults between 24 and 35 years of age were immunized with the mRNA-1273 vaccine formulating mRNA molecules encoding for the full length of the spike (S) protein of the original SARS-CoV-2 strain. The participants were immunized during the spring of 2021 as part of the national (United Kingdom) vaccination program. Demographic details of the participants can be found in Table S1. Influence or association of sex and/or gender was not possible due to low n number of the study and the variability of the participants to such a response as is vaccination. Ethnicity, ancestry, race and socioeconomic status of the participants was not recorded as this information was not part of the ethics study submission. Informed consent was asked of all participants prior to the start of the study. Participants were surveyed for previously known SARS-CoV-2 infection and other relevant (immune-related) co-morbidities at the start and at the end of the study. Ethical approval was obtained from the East Midlands - Leicester Central Research Ethics Committee, under REC reference no. 21/EM/0064. Additionally, COVID-19 infection samples were collected from SARS-CoV-2 positive patients at Frimley and Wexham Park hospitals during 2020 (consented under UK London REC no. 14/LO/1221).^8^

Whole blood, serum, peripheral blood mononuclear cells (PBMCs), saliva and nasal wash were taken for a variety of readouts including measurement of antigen-specific antibody levels by ELISA, antibody blocking capacity, multicolor spectral flow cytometry for immunophenotyping from whole blood and PBMCs, single-cell RNA sequencing (scRNA-seq) and bulk B cell receptor (BCR) sequencing at various time points (Figure 1A). A baseline time point (Day −1) was scheduled 24h prior to initial vaccination which happened on day 0 with subsequent timepoints happening every Monday, Wednesday and Friday for 3 weeks: time points D+2 to D+26 (Figure 1A). The second vaccine dose visit occurred 8 weeks after the first dose with a baseline time point (week 8 or W8) taken before this second immunization. Additional two post-second dose timepoints at 10 (W10) and 12 (W12) weeks after initial vaccination, or 2 and 4 weeks after second dose respectively, were taken, with a final time point 6 months (M6) after initial vaccination (Figure 1A). Participants were screened for previous SARS-CoV-2 reactivity (either through infection or cross-reactivity) with two participants (P6 and P14) showing anti-RBD IgG antibodies at baseline (Figure S1A). These two participants were excluded from subsequent analysis.

For single-cell transcriptomics, the following time points were selected for analysis: D-1 as baseline, D+5 which was prior to class-switched B cell expansion (Figures S6B and S6C), as well as D+9 and D+12 which coincided with changes in IgG and IgA class-switched antibodies at the secreted and transcriptional levels (Figure S1B; Figures S3C and S3D). Cells from W8 and W10 were also analyzed to characterize secondary B cell responses to the vaccine.

### Method details

#### Whole blood, PBMC and serum isolation

Whole blood was collected in sodium heparin tubes (455051, Greiner Bio-One) and serum SST tubes (456018, Greiner Bio-One). 100mL of non-coagulated whole blood from heparin tubes was kept separately for whole blood *ex vivo* immune phenotyping. Non-coagulated whole blood from heparin tubes was diluted 1:1 in PBS (14040-091, Gibco) with 2% heat-inactivated fetal bovine serum (FBS, FCS-SA/500–22512, Labtech). Density gradient centrifugation using SepMate tubes (85450, STEMCELL Technologies) was used to isolate peripheral blood mononuclear cells (PBMCs) according to the manufacturer’s instructions. PBMCs were counted and viability estimated using a trypan blue exclusion assay. Cells were then resuspended at 10^7^ viable cells per mL in FBS containing 10% DMSO (20688, ThermoScientific) and stored in liquid nitrogen. Serum SST tubes were centrifuged at 1200g for 10 min at room temperature, then serum was aliquoted into cryovials and stored at −70°C.

#### Quantification of RBD-specific antibody titers

Anti-RBD IgM/A/G ELISAs were performed based on a published plasma-based method.^101^ High-binding 96 well plates (9018, Corning for COVID Vaccine samples and 442404, ThermoFisher for COVID patient samples due to plastic shortages) were coated with 100μL of 2μg/ml of RBD (ab273065, Abcam) in PBS and incubated at 4°C overnight. Plates were then washed three times with 0.01% Tween 80 (P5188-100ML, SigmaAldrich) in PBS (PBS-T) and blocked with 200μL of 3% milk (84615.0500, VWR) in PBS-T for between 1 and 4 h. Blocking buffer was removed and plates tapped dry, and 100mL of diluted samples/controls added. Serum was diluted at either 1:20, 1:60, 1:100, 1:300, 1:900 or 1:2700 with PBS to have the OD fall within the linear range of the standard curve/positive controls and diluted once more 1:3 on the plate with PBS-T with 1% milk powder. Positive controls for corning plates were loaded at initial concentrations of 0.2 ng/μL for IgM (Ab01680-15-0, Absolute Biotech, clone CR3022), 0.602 ng/mL for IgA (Ab01680-16-0, Absolute Biotech, clone CR3022), 0.2 ng/μL for IgG (ab273073, Abcam, clone CR3022) and for maxisorb at 0.06 ng/mL for IgM, 0.20067 ng/mL for IgA, 0.06 ng/μL for IgG, and then serially diluted 1:2 four times. All samples and positive controls/standard curve points were run in duplicate. Each plate also contained two blanks and negative controls from pre-pandemic serum samples. Samples were incubated for 2 h at room temperature, washed 3 times and 50 mL of HRP-conjugated detection antibody (IgM: A18835, ThermoFisher; IgA: A0295-1ML, Sigma-Aldrich/Merk; IgG: A18817, ThermoFisher) added and incubated for 1 h (RT), then washed 3 times and loaded with 100 mL of OPD (#11879250, FisherScientific) to reveal for 15 min and finally with 50 mL of 3M Hydrochloric acid (HCl) used to stop the reaction. Plates were read on a SpectraMax iD3 plate reader (Molecular Devices) at 490 nm. Sample values were standardized against the blank control by subtraction and antibody titers interpolated in GraphPad Prism (v9) and multiplied by their dilution factor.

#### Recombinant S1-specific antibody production and reactivity test

##### S1-specific antibody expression

Variable region sequence (A8 clone) from the overlapped single and bulk dataset were cloned into pFUSE IgG1, IgG2, IgG3, kappa, and lambda expression constructs and transiently expressed using the Expi293F expression system (Thermo Fisher Scientific). Expi293F cells were thawed, expanded in Expi293 Expression Medium under standard conditions (37°C, 8% CO_2_, 125 rpm), and passaged at least three times to ensure optimal viability and growth. Transfections were performed at a cell density of 3 × 10^6^ viable cells/mL using ExpiFectamine 293 reagent, with 0.8 μg total plasmid DNA per well at a 1:2 heavy-to-light chain ratio. At 18–22 h post-transfection, Transfection Enhancers 1 and 2 were added according to the manufacturer’s protocol. Cultures were maintained for 5–7 days before harvesting for antibody production analysis.

##### Recombinantly produced antibody reactivity to SARS-CoV-2 spike protein

To assess the binding activity of expressed antibodies to the SARS-CoV-2 spike (S) protein, an indirect ELISA was performed. High-binding 96-well plates (Corning Costar, #3361) were coated overnight at 4°C with 100 μL per well of recombinant SARS-CoV-2 spike protein (1 μg/mL in PBS; R&D Systems, #11058-CV). The following day, plates were washed three times with PBS containing 0.05% Tween 20 (PBS-T), then blocked with 200 μL per well of PBS supplemented with 10% heat-inactivated FBS for 90 min at room temperature.

After blocking, 50 μL per well of diluted NIBSC Anti-SARS-CoV-2 Antibody Diagnostic Calibrant (21/338; 1:12,800 to 1:102,400) and supernatants containing expressed A8 antibody variants (IgG1, IgG2, IgG3; paired with either kappa or lambda light chains; 1:3 dilution) were added in duplicate. Plates were incubated for 90 min at room temperature with orbital shaking (200 rpm).

Plates were then washed, and 50 μL/well of biotin-conjugated goat anti-human IgG secondary antibody (Millipore, AP112B) diluted 1:20,000 in blocking buffer was added, with incubation for 90 min at room temperature in the dark. Subsequent incubation and detection were performed with 50 μL/well Thermo Scientific Pierce High Sensitivity Streptavidin-HRP (1:20,000 dilution) for 20 min and 50 μL/well TMB substrate (Sigma-Aldrich, T4444) until adequate color development to avoid saturation, or up to maximum of 20 min. The reaction was stopped with 50 μL of 0.16 M sulfuric acid per well.

Absorbance was measured at 450 nm with background correction at 540 nm using a Spectra Max iD3 plate reader (Molecular Devices). Sample reactivity was assessed relative to the standard calibrant and background controls.

#### Bulk B cell receptor (BCR) library generation

3mL of whole blood at each timepoint was taken into Tempus Tubes (4342792, Applied Biosciences) and RNA was extracted according to manufacturer’s instructions. Bulk Immunoglobulin repertoire libraries were prepared as previously described.^8^ Briefly, a 5′ template switch transcription was performed to incorporate Unique Molecular Identifiers (UMIs), followed by two rounds of polymerase chain reaction (PCR). The first PCR stepped-out to add a primer landing site at the 5′ end, facilitating the step-out addition of donor identifier barcodes at the 5′ end in PCR2 for multiplexing. The reverse primers were designed to nest in the constant regions with step-out multiplex identifiers added in PCR2. Libraries were sequenced on a Pacific Biosciences (PacBio) Sequel IIe system at the Liverpool Center for Genomic Research. Quality control, data cleaning and removal of multiplicated UMIs were performed as previously described.^8^ Our bulk BCR repertoire dataset contained a total of 3,778,590 sequences.

#### Blocking assay

A V-PLEX SARS-CoV-2 panel 30 (ACE2) kit (K15635U, Meso Scale Diagnostics) was used according to the manufacturer’s instructions to measure the capacity of serum samples to block the binding of variants of SARS-CoV-2 to the ACE2 receptor. A dilution of 1:50 was used for all serum samples.

#### Whole blood *ex vivo* immune phenotyping

100mL of non-coagulated fresh whole blood from heparin tubes was stained with additional 100μL staining buffer the surface antibodies described in the key resources table (indicated as “whole blood for analyzer”) at room temperature in the dark for 30 min. Then 2mL of red blood cell lysis and fixation buffer (00-5333-54, eBioscience) were added and incubated at room temperature in the dark for 25 min. Fixed samples were centrifuged at 800g for 5 min at room temperature. Finally, cells were washed with 3mL of FACS buffer (PBS 2% FBS and 0.2mM EDTA (15575-038, Invitrogen)) and centrifugation at 800*g* for 5min at room temperature. Samples were acquired in a Digital LSR II flow cytometer (BD Biosciences). Flow cytometric data were analyzed using FlowJo software (v10.8.1). Antibodies for whole blood staining were previously specially titrated for this staining. A higher concentration than that used for PBMCs was chosen as antibody concentration used for PBMCs yielded poor staining. This is likely due to the presence of red blood cells in great numbers in whole blood samples.

#### Antigen-specific B cell *ex vivo* phenotyping

Vaccine-derived antigen-specific B cells were identified by tagging them with the subunit 1 (S1) of the spike protein (S) of the ancestral SARS-CoV-2 and the receptor-binding domain (RBD), a domain contained within the S1. Biotin-conjugated S1 was coupled with two fluorochrome-labelled streptavidin to form two different S1-fluorochome conjugates. Biotin-S1 (793806, Biolegend) was conjugated with streptavidin-BV421 (405225, Biolegend) and streptavidin-APC (405207, Biolegend) separately, in PBS at a ratio of 1:6 (streptavidin-fluorochrome:biotin-S1). Biotin-conjugated RBD (793904, Biolegend) was conjugated with streptavidin-BUV737 (612775, BD Biosciences) in PBS at ratio of 1:4 (streptavidin-fluorochrome:biotin-RBD). A decoy conjugate consisting of only biotin coupled with a streptavidin-fluorochrome complex was constructed by mixing D-biotin (B-4501, SigmaAldrich) and streptavidin-PE-Cy5 (405205, Biolegend) at a ratio of 1:40 (streptavidin-fluorochrome:free D-biotin). These conjugates were incubated under agitation at 4°C for at least one hour. 5 × 10^6^ PBMCs were stained per sample (for each participant and timepoint analyzed). PBMCs were thawed at 37°C until a small amount of ice remained, 500μL of pre-warmed 37°C FBS was added, samples transferred to a 15mL falcon and diluted to 10mL with complete RPMI (10% heat-inactivated FBS and 1% penicillin/streptomycin (P0781, SigmaAldrich), centrifuged at 500g for 8 min at 4°C and washed once with PBS by centrifuging at 500g for 8 min at 4°C. Cells were incubated in 200μL of LIVEDEAD fixable blue (L23105, Invitrogen) diluted 400-fold in PBS together with FcR blocking agent (130-059-901, Miltenyi) at 4°C in the dark for 30 min. Cells were then washed with FACS buffer and centrifuged at 500g for 8 min 200μL of 5mM D-biotin FACS buffer (D-biotin FACS buffer) containing 5ng of Biotin-PE-Cy5 (decoy) was added and incubated for 30 min in the dark at 4°C. Following incubation with the decoy, cells were washed twice with D-biotin FACS buffer by centrifuging at 500g for 8 min. Cells were then stained with the antigen probe cocktail (0.5mg/samples for each S1 construct and 0.25mg/sample for the RBD construct) and surface antibodies (described in the key resources table as “PBMCs for analyzer”) in 200mL of D-biotin FACS buffer and left in the dark at 4°C for one hour to incubate. Tubes containing the cells were agitated every 20 min during this incubation period to ensure maximal staining. Cells were then washed with D-biotin FACS buffer and centrifuged at 500g for 8 min before 200μL of IC Fixation Buffer (00-8222-49, eBioscience) were added left to incubate for 20 min at 4°C in the dark. Following fixation, cells were washed twice with FACS buffer by centrifuging at 500g for 8 min and resuspended in FACS buffer for acquisition. Samples were acquired in a CytekTM Aurora cytometer (5 lasers) using SpectroFlo (v3.0.3) with automated unmixing. Flow cytometric data were analyzed using FlowJo software (v10.8.1). In total we analyzed 16,293,828 S1^-^ and S1^+^ B cells over all donors and timepoints considered in this flow cytometry analysis.

#### Single-cell transcriptomic library generation

Vaccine-derived antigen-specific B cells were identified by tagging them with the subunit 1 (S1) of the spike protein (S) of the ancestral SARS-CoV-2 and the RBD. Biotin-conjugated S1 was coupled with two fluorochrome/oligomer dual-labelled streptavidin to form two different S1-fluorochome/oligomer conjugates. In this way, antigen-specific B cells were sorted via flow cytometry assisted sorting (FACS) using the fluorochromes and sequenced using single cell technologies using the oligomer sequence with a posterior bioinformatic identification of antigen-specific B cells. Biotin-S1 (793806, Biolegend) was conjugated with TotalSeq streptavidin-PE (405261, Biolegend) and TotalSeq streptavidin-APC (405283, Biolegend) separately, in PBS at a ratio of 1:6 (streptavidin-flurochrome+oligomer:biotin-S1). Biotin-conjugated RBD (793904, Biolegend) was conjugated with streptavidin-oligomer (405271, Biolegend) in PBS at ratio of 1:4. A decoy conjugate consisting of only biotin coupled with a streptavidin-fluorochrome complex was constructed by mixing D-biotin and streptavidin-FITC (405201, Biolegend) at a ratio of 1:40 (streptavidin-FITC:free D-biotin). These conjugates were incubated under agitation at 4°C for at least one hour. 5 × 10^6^ PBMCs were stained per sample (for each participant and timepoint analyzed). PBMCs were thawed at 37°C until a small amount of ice remained, 500μL of pre-warmed 37°C FCS was added, samples transferred to a 15mL falcon and diluted to 10mL with cRPMI and counted, washed once with PBS by centrifuging at 500g for 8 min at 4°C. Cells were incubated in 200μL of staining mix containing Zombie NIR (423105, Biolegend), both S1 construct (S1-APC+oligomer, S1-PE+oligomer) the RBD construct (RBD-FITC), the FITC decoy (D-biotin-FITC) and the extracellular antibodies described in the key resources table as “PBMCs for sorting” at 4°C in the dark for 1 h.

Cells were then washed with FACS buffer and centrifuged at 500g for 8 min at 4°C. Samples were acquired and sorted in a BD FACSAria Fusion. All events were gated for lymphocytes based on FSC/SSC, singlets and living cells. B cells were identified as CD19^+^ cells, decoy negative B cells further selected, and S1^+^ B cells detected by double PE and APC staining (Figure S2d). Four populations were sorted: S1^+^ B cells (CD19^+^PE-S1^+^APC-S1^+^), S1^-^ B cells (CD19^+^PE-S1^+^APC-S1^+^), CD19^−^ lymphocytes and innate immune cells. Innate immune cells were gated on the same basic criteria and size selected based on size with CD14 on z axis to aid size selection. Samples across time points for each donor were processed in the same batch and sorted cell populations from each time point were pooled as follows: 60% S1^-^ B cells, 20% CD19^−^ lymphocytes, 20% innate immune cells. Hashtags were added individually for each day’s sample (Biolegend 394661, 394663, 394665, 394667, 394669, 394671) to allow bioinformatic demultiplexing using the feature barcode sequencing reads. A pool of all S1^+^ B cells, 60% S1^-^ B cells, 20% CD19^−^ Lymphocytes, 20% Innate cells was run in 4 10X reactions lanes for 5000 cells in each reaction. Cells were centrifuged (500g 5 min at 4°C) and resuspended in PBS with non-acetylated BSA to the desired concentration and run on the 10X chromium controller utilizing the Chromium Next GEM Single cell 5′ reagent kit v2 (Dual Index) (document number: CG00030 Rev F) producing the GEX, VDJ sequencing and cell surface protein libraries according to the manufacturer’s instructions. Libraries were sequenced on a HiSeq2000 or NovaSeq X Plus Series (PE150) at 50,000 reads per cell for GEX libraries and 5,000 read per cell for VDJ/Cell surface protein libraries by Novogene.

#### BCR repertoire data analysis

BCR sequences were annotated for immunoglobulin VDJ gene usage using IMGT/HighV-Quest.^96^ Clonotype clustering was performed as previously described^8^ by calculating Levenshtein distance pairwise between CDRH3 nucleotide sequences. The resultant distance matrix was hierarchically clustered, and branches were cut at 0.05 to define clones. Physicochemical properties were calculated using the R Peptides package (v2.4.4).^102^ Clonal diversity was calculated using the Gini coefficient, which measured the evenness in the distribution of clone size. We used the transformation (1 – Gini coefficient) as a measure of clonal diversity. For separating clonotypes into Low and High SHM groups, we used a *IGHV* germline identity cut-off of 99%; we observed that this effectively separate bimodal distributions of germline identity in all the isotypes (Figure S3B).

BCR lineage trees were constructed using the BrepPhylo^8^ package (v0.4.2). Briefly, a maximum parsimony tree was first constructed for each clone using the dnapars executable in the phylip package,^103^ using the IMGT-gapped V-gene nucleotide sequences as input. All clones with at least 3 sequences were considered. From these trees we calculated, for each observed sequence in a given clone, its distance to the annotated germline gene. This tree-based distance from the germline measures the extent of mutation accumulation for the given sequence.^8^ We further analyzed the reconstructed lineage trees to identify class-switch events, i.e., branches in the tree which connect BCR sequences of different isotypes. In BrepPhylo we previously implemented routines to prune the dnapars trees (which were built using only V gene sequences) to remove edges which implicate CSR events that violate the physical order of constant region genes in the human IGH locus, and build minimum spanning arborescence tree of the pruned data using Edmond’s algorithm.^8^ These trees were used to identify and quantify CSR events between any pairs of isotypes in the data.

#### Single-cell transcriptomic data analysis

##### Data preprocessing and clustering

Matching 10X genomics gene expression, BCR and feature barcode/cell surface protein libraries were processed through CellRanger multi version 6.1.2. The following reference genome versions were downloaded from the cellranger website for sequence alignment and annotation: refdata-gex-GRCh38-2020-A (for gene expression libraries) and refdata-cellranger-vdj-GRCh38-alts-ensembl-5.0.0 (for BCR libraries). For data processing steps outlined below, the R package Seurat (v4.3.0)^99^ was used unless otherwise stated. The raw read count matrix for each library was first preprocessed to collapse individual genes belonging to each of the following groups of genes to eliminate donor-specific variations from dominating downstream cell clustering: immunoglobulin variable (V) genes (gene name patterns matching regular expression “ˆIG[HKL]V[0–9]”), diversity (D) genes (“ˆIG[HKL]D[0–9]”), joining (J) genes (“ˆIG[HKL]J[0–9]”), ribosomal genes (“ˆRP[LS]|ˆMRP[LS]”), individual HLA class Ia genes (“ˆHLA-[ABC]$”), HLA class Ib genes (“ˆHLA-[EFG]$”), HLA class II genes (“ˆ HLA-D”). Read counts mapped to genes belonging to these gene groups were collapsed into separate metagenes and replaced the individual genes listed therein. The percentage of reads mapped to mitochondrial genes (“ˆMT-|ˆMTRNR”) per-cell were calculated and appended as cell metadata. Cells with transcripts mapped to between 200 and 4000 distinct genes & a mitochondrial read percentage below 15% were retained for analysis. The SCTransform^104^ protocol implemented in Seurat was applied for read count normalization, with the mitochondrial read percentage modeled as a covariate to remove variation attributable to this factor. Genes at the immunoglobulin, T cell receptor, HLA and mitochondrial loci were removed from the list of variably expressed genes prior to dimensionality reduction and clustering to remove their impact in driving the definition of cell clusters. Dimensionality reduction was performed using principal component analysis (PCA) using this pruned list of variably expressed genes. A k-nearest neighbor graph was constructed using the first 14 principal components, and cell clustering was performed on this graph using the FindClusters function with the resolution parameter of 0.5. B cells were identified by examining CD20 (*MS4A1*) expression across these clusters. The B cell clusters were subsetted from the data and analyzed separately from the non-B cells. For B cells, cell labels were assigned per cell by transferring from our previously published scRNA-seq atlas of peripheral B cells,^16^ using the TransferData protocol implemented in Seurat, by projecting the PCA structure of the reference to this dataset to classify cells based on similarity in this (projected) PCA space.

##### Demultiplexing

Time point specific hashtags were demultiplexed following the HTODemux protocol implemented in Seurat, where hashtag read counts were normalized using centered log-ratio transformation and thresholds for demultiplexing were determined using the 95^th^ percentile of the normalized read-count distribution as the cut-off to define hashtag positive and negative cells.

##### Identifying S1-specific B cells

Initial analysis of the raw read count data for feature barcodes corresponding to the S1 or RBD baits indicated that signals for the three antigen-specific TotalSeq feature barcodes (PE Strep-S1, APC Strep-S1, and Strep-RBD) vary greatly, with a lack of signals corresponding to PE Strep-Spike in comparison with TotalSeq APC Strep-Spike. TotalSeq Strep-RBD raw counts suffered from significant background noise. We reasoned that an explicit model of background and true signals in these read counts *was* necessary for confident identification of S1-specific B cells. We therefore trained a totalVI^98^ model using our paired gene expression and feature barcode libraries. TotalVI explicitly model background and foreground distributions for feature barcode libraries^98^; here we used this to correct for substantial noise in our raw read counts corresponding to the antigen-specific barcodes. We used the get_protein_foreground_probability() function to obtain the corrected antigen-specific signal as a probability score between 0 and 1; the probability distributions for TotalSeq APC Strep-Spike and Strep-RBD were bimodal with two peaks close to 0 and 1. We therefore used these probability scores to identify S1- and RBD-specific B cells, using a cut-off at 0.5 for identifying antigen-positive B cells.

##### Sterile and productive immunoglobulin transcript analysis

We used sciCSR^18^ (v0.3.3) to analyze BAM alignments of the gene expression libraries, for identifying sterile transcripts at the immunoglobulin heavy-chain gene locus. Sterile transcripts are immunoglobulin transcripts which lack coding information for the V, D and J gene segments, and instead start at genomic positions 5′ to the beginning of every constant (C) region genes. Sterile transcription is an indication of B cells poised for class-switching.^18^ Using sciCSR we obtained a read count matrix for all heavy-chain sterile transcripts, which were subsequently log-normalized, and the expression levels of these transcripts were visualized using dot plots. For productive transcripts, we integrated the matching single-cell BCR libraries with the gene expression data based on matching cell barcode. In cases where multiple heavy and/or light chain transcripts share the same cell barcode, these cells were flagged and only the transcript with the highest unique molecule identifier (UMI) count was retained for merging with the cell metadata. Columns in the filtered contig annotation output from cellranger corresponding to V, D, J, C genes were retained in the merged cell metadata for BCR isotype-based analysis presented here. V region germline identity was obtained via IgBLAST (v1.19.0) analysis against the set of germline immunoglobulin VDJ alleles obtained from IMGT (accessed 28-Jul-2022). We have applied the TIgGER package^100^ (v1.1.0) to assess the possibility of donor-specific VDJ alleles that would bias this analysis, but did not identify any novel alleles from the sequencing data. Physicochemical properties of CDRH3 amino acid sequences were calculated using the Peptides package^102^ (v2.4.4) through BRepertoire.^97^

#### Bulk and single-cell BCR data integration

We identified exact overlaps in terms of CDRH3 amino acid sequence between the bulk and single-cell BCR annotated sequence data, and utilize the antigen specificity labeling information in the single-cell data to annotate clonotype lineages sampled in the bulk data. This approach capitalizes on the deep sampling of the antibody repertoire in the bulk libraries while filling in antigen specificity information missing from this data. For single-cell data, we considered only sequences with cell barcodes where exactly one heavy and one light chain productive transcript were observed. The CDRH3 amino acid sequences of clonotypes from the bulk data were scanned for exact matches with the single cell data, to extract a list of donor and clonotype identifiers (hereafter “overlapping clonotypes”). This procedure considered only heavy-chain sequences from both the bulk and single-cell data. An overlapping clonotype was annotated as S1^+^ if at least one of the heavy-chain sequences in this clonotype that originated from the single-cell data corresponds to a B cell with S1-binding probability score (see subsection “Identifying S1-specific B cells” under “Single-cell transcriptomic data analysis”) greater than 0.5. CSR-aware arborescence trees were reconstructed for every overlapping clonotype using BRepPhylo^8^ (v0.4.2) using the identical procedure as detailed above for bulk BCR repertoire analysis. For each edge in the tree we annotated whether the edge connects sequences of different isotypes to identify class-switch events.

To systematically compare the class-switch features of the S1^+^ and S1^-^ trees, we enumerated the proportion of branches with class-switch events for each clonotype tree.

### Quantification and statistical analysis

Statistical analysis was performed in the R statistical computing environment (v4.2.0). Data visualization was produced using the ggplot2 package (version 3.4.1). Bootstrap sampling was performed using the boot R package (version 1.3–28). We considered the 2.5% and 97.5% percentiles of the distribution of the bootstrapped statistic as its 95% confidence interval. Mixed effect models were fitted using the lmerTest R package (version 3.1–3) and model estimates were optimized according to the restricted maximum likelihood (REML) criterion. Wherever possible, mixed effect models were fitted with donor as random effect and the fixed effect was set to be the variable for which the desired contrasts were analyzed. Wherever relevant, sample sizes were indicated in the results section, as well as in figure legends. All statistical tests used and the definition of center and dispersion measures in error bars included in the figure legends wherever relevant.

### Additional resources

The multi-omics data generated in this study can be downloaded via identifiers and links given in the Key Resource Table. The processed data can also be viewed and queried on a dedicated web-based viewer at https://fraternalilab.cs.ucl.ac.uk/CovVaxBcells/.

## Supplemental Information

Document S1. Figures S1–S14 and Tables S1 and S2

Document S2. Article plus supplemental information
