# Supplementary material for: Human primary antibody response to vaccination follows a partially sequential class-switching program with a checkpoint at IGHG2
Source: Cell Rep Med. 2026 Jun 4;7(7):102848. doi: 10.1016/j.xcrm.2026.102848 (PMC13400142; doi:10.1016/j.xcrm.2026.102848)
Supplement: Document S1. Figures S1–S14 and Tables S1 and S2 [file mmc1.pdf]

Cell Reports Medicine, Volume *7*

Supplemental information

Human primary antibody response to vaccination follows a partially sequential class-switching program with a checkpoint at *IGHG2*

Guillem Montamat-Garcia, Joseph C.F. Ng, Alexander T. Stewart, Emma Sinclair, Benedicta B. Mensah, Yan Hui Giam, Paul Blair, Diana Kateregga, Amir Gander, David Kipling, Dongjun Guo, Lutecia Servius, Christopher J.M. Piper, Zara Baig, Franca Fraternali, Claudia Mauri, and Deborah K. Dunn-Walters

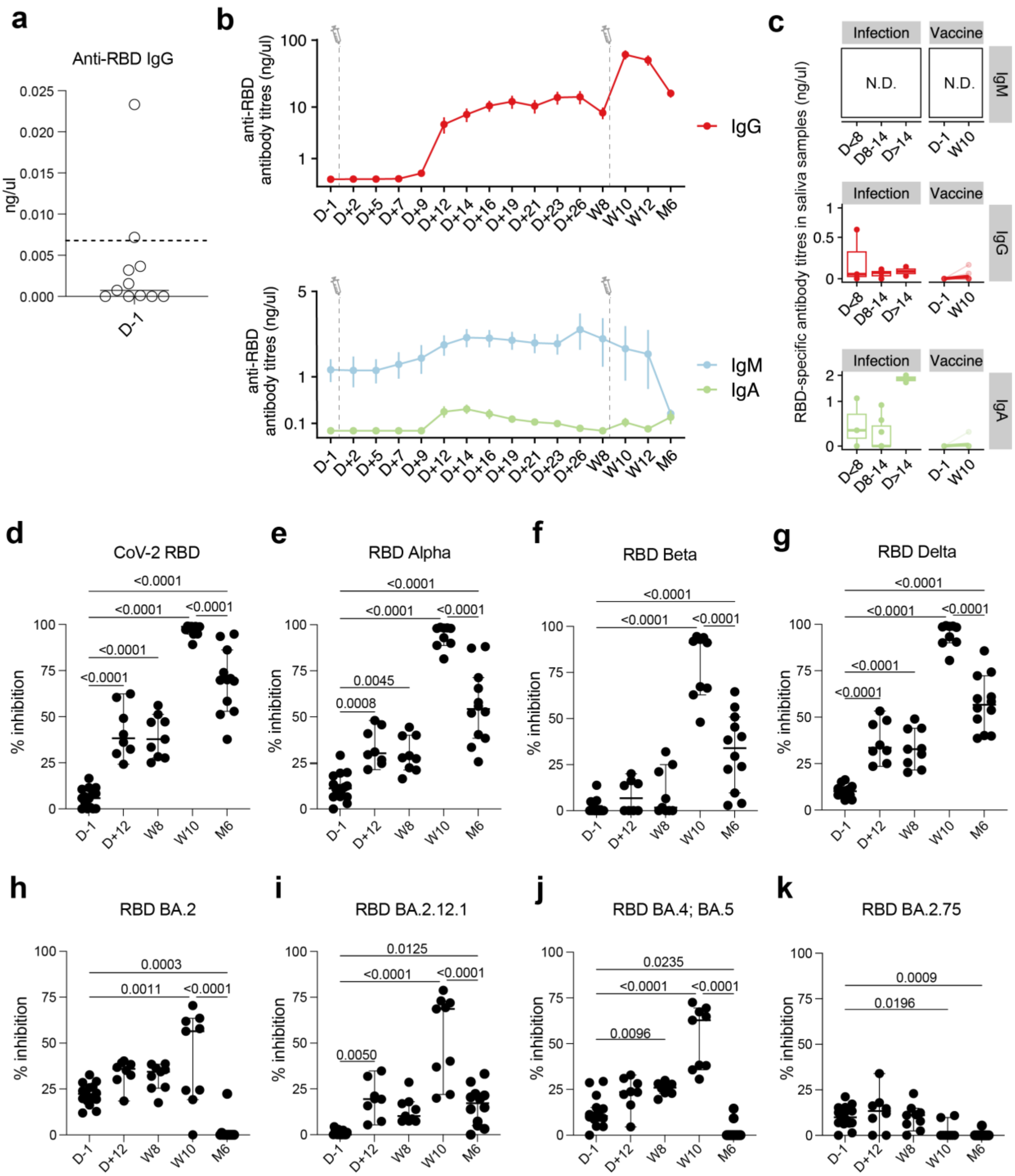

Figure S1. SARS-CoV-2 vaccination against ancestral strain induces antibody titer changes and impairs long term blocking capacity of RBD-omicron. Related to Figure 1.

(a) Anti-Receptor Binding Domain (RBD) IgG antibody titers measured at D-1 using ELISA. Data points from two outlier donors with previous SARS-CoV-2 exposure

(P06 and P14) were highlighted separately. Dotted line indicates ELISA detection limit.

(b) Changes in serum RBD-specific IgG, IgA, and IgM levels across time. Trend line represents mean values per time point across donors with available data (n=12); error-bars depict standard error of means.

(c) Antibody titers of IgM, IgG, and IgA in saliva samples from hospitalized SARS-CoV-2 infected patients (n=9, Stewart, Sinclair, Ng et al. Front Immunol 2021) ("Infection") and this vaccination cohort ("Vaccine"). For the Infection cohort, time points denote number of days since hospitalization. Data points denote measurements from individual donors. Error bars indicate the range observed over all observations.

(d-k) Percentage inhibition of binding to the ACE2 receptor against different SARS-CoV-2 strains (d) CoV-2 (Wu-1/ancestral), (e) Alpha, (f) Beta, (g) Delta, and omicron stains (h) BA.2, (i) BA.2.12.1, (j) BA.4;BA.5, and (k) BA.2.75 of serum collected at different timepoints during the vaccine response. n=8-15. Error bars indicate standard error of means. One way ANOVA with multiple comparison using Sidak's correction was used to calculate the shown p-values for each significant paired comparison.

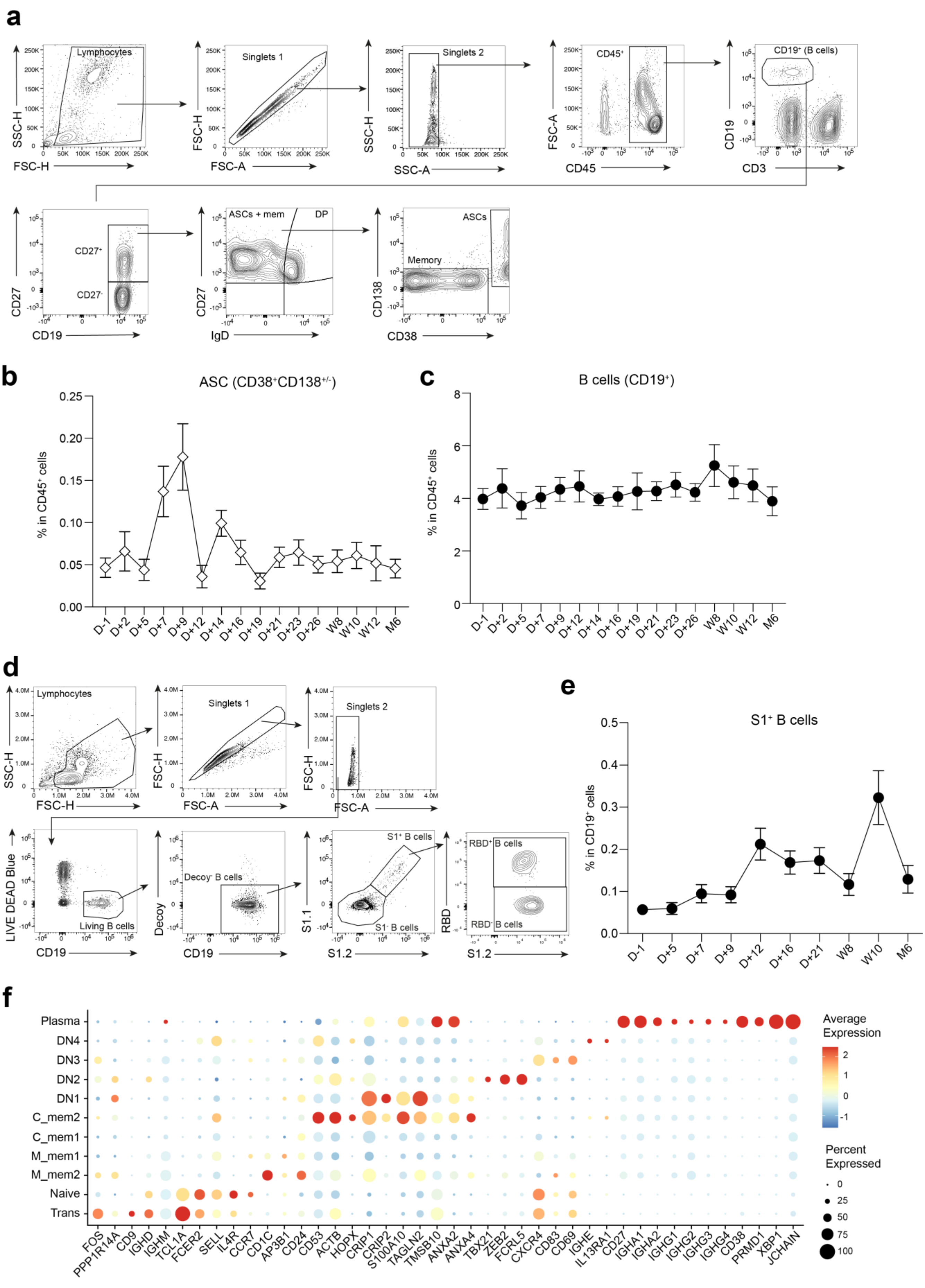
a
Lymphocytes
SSC-H
FSC-H
Singlets 1
FSC-H
FSC-A
Singlets 2
SSC-H
SSC-A
CD45+
FSC-A
CD45
CD19+ (B cells)
CD19
CD3
CD27+
CD27-
CD27
CD19
ASCs + mem
DP
CD27
IgD
ASCs
Memory
CD138
CD38
b
ASC (CD38+CD138+/-)
% in CD45+ cells
D-1
D+2
D+5
D+7
D+9
D+12
D+14
D+16
D+19
D+21
D+23
D+26
W8
W10
W12
M6
c
B cells (CD19+)
% in CD45+ cells
d
Lymphocytes
SSC-H
FSC-H
Singlets 1
FSC-H
FSC-A
Singlets 2
FSC-H
FSC-A
LIVE DEAD Blue
Living B cells
CD19
Decoy- B cells
Decoy
CD19
S1+ B cells
S1- B cells
S1.1
S1.2
RBD+ B cells
RBD- B cells
RBD
S1.2
e
S1+ B cells
% in CD19+ cells
D-1
D+5
D+7
D+9
D+12
D+16
D+21
W8
W10
M6
f
Plasma
DN4
DN3
DN2
DN1
C_mem2
C_mem1
M_mem1
M_mem2
Naive
Trans
FOS
PPP1R14A
CD9
IGHD
IGHM
TCL1A
FCER2
SELL
IL4R
CCR7
CD1C
AP3B1
CD24
CD53
ACTB
HOPX
CRIP1
CRIP2
S100A10
TAGLN2
TMSB10
ANXA2
ANXA4
TBX21
ZEB2
FCRL5
CXCR4
CD83
CD69
IGHE
IL13RA1
CD27
IGHA1
IGHA2
IGHG1
IGHG2
IGHG3
IGHG4
CD38
PRMD1
XBP1
JCHAIN
Average Expression
Percent Expressed

Figure S2. The B cell compartment and S1$^+$ B cells in flow cytometry and single-cell BCR sequencing data. Related to Figure 1.

(a) Representative plots showing the gating strategy for identification of total CD19$^+$ B cells (CD19$^+$), and circulating antibody secreting cells (ASCs, CD19$^+$CD27$^+$IgD-CD38$^+$CD138$^{+/-}$) in whole blood.

(b) Changes in CD38$^+$ antibody secreting cells (ASCs) as percentage of CD45$^+$ cells across time. n=13; error-bars depict standard error of means.

(c) Frequency of total B cells (CD19$^+$) as a proportion of CD45$^+$ cells during vaccine response. n=6-13; error-bars depict standard error of means.

(d) Representative plots showing the gating strategy for identification of antigen-specific (S1$^+$) B cells through flow cytometry analysis or fluorescence-activated cell sorting (FACS) and subsequent single cell RNA sequencing.

(e) Frequency of S1$^+$ total B cells (CD19$^+$) as a proportion of CD19$^+$ cells during vaccine response. n=9; error-bars depict standard error of means.

(f) Dotplot displaying key marker genes for 11 B cell subpopulations annotated in the scRNA-seq data (S1$^+$ and S1$^-$ B cells) for n=35,426 B cells.

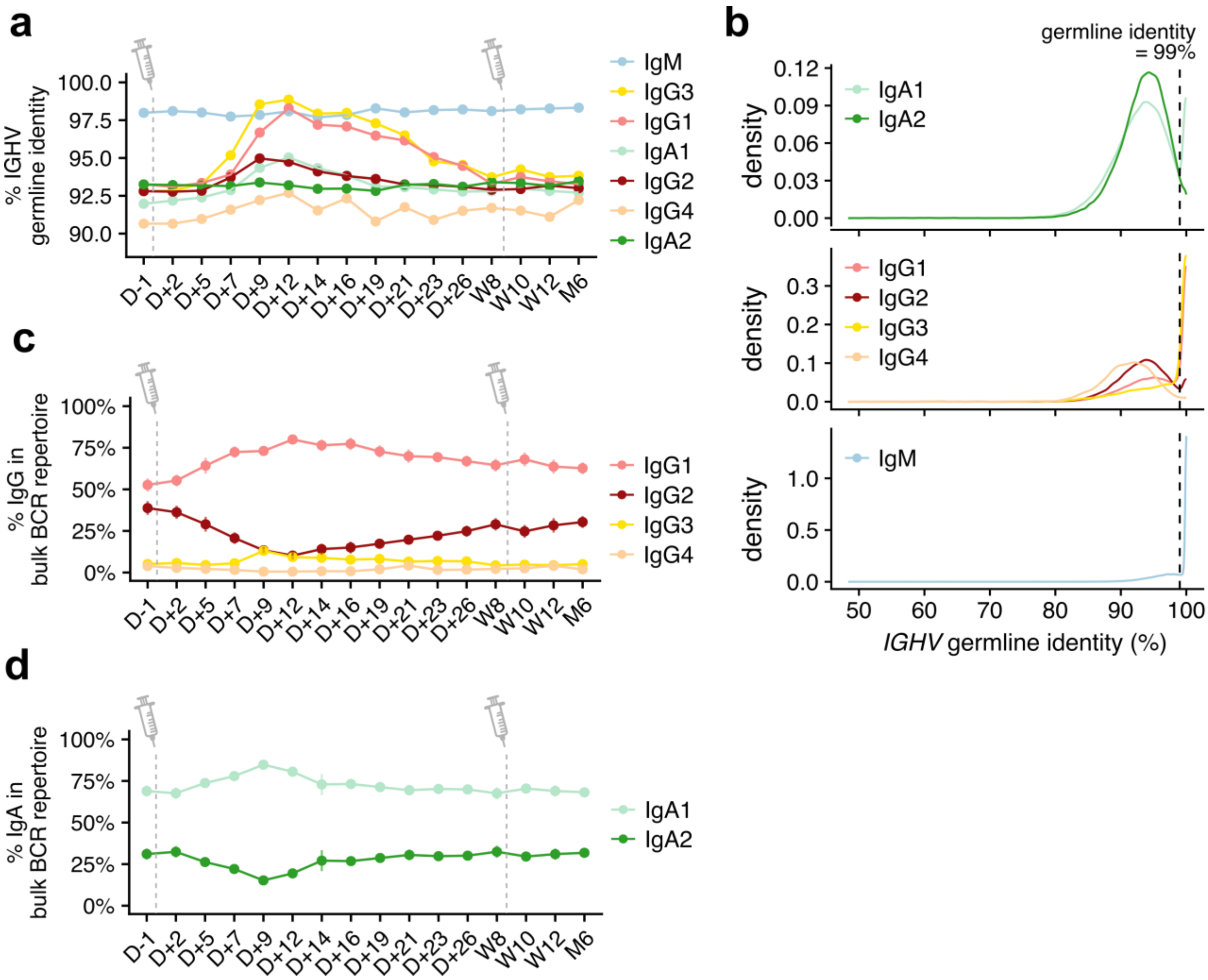

Figure S3. Evolution of *IGHV* germline identity and *IGHC* subtype-proportion in bulk BCR sequencing data. Related to Figure 1.

(a) Changes in BCR sequence identity to germline *IGHV* gene divided by isotype subclass across the vaccination time-course. Trend-lines display mean values per time point across all donors with available data (n=9 donors); error-bars depict standard error of means.

(b) Distributions of *IGHV* germline identity divided by isotype subclass. Dotted line depicts the cutoff of 99% used to define low versus high SHM sequences analyzed separately for CSR events.

(c-d) Changes in subclass percentage distribution based on bulk B cell receptor (BCR) repertoire data for (c) IgG isotypes and (d) IgA isotypes across the vaccination time-course. Trend-lines display mean values per time point across all donors with available data (n=9 donors); error-bars depict standard error of means.

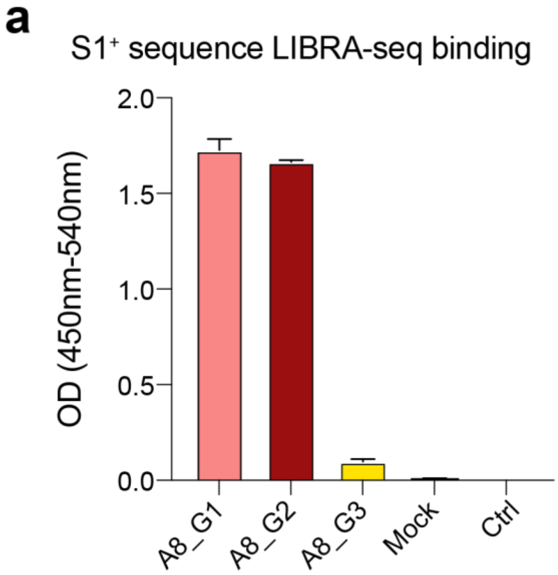

Figure S4. Validation of S1⁺ BCR discovered via LIBRA-seq. Related to Figure 1.

(a) A selected S1⁺ clone (A8) generated by the LIBRA-seq was cloned and produced in three different constant regions (IgG1, IgG2 and IgG3) and tested for binding to SARS-CoV-2 (Wu-1/ancestral) spike protein by ELISA without antibody concentration correction. Mock is the supernatant of mock transfected cells without any plasmid. Negative control (Ctrl) shows a non-spike specific IgG1 cloned and produced sequence. Bar plot shows mean ±standard deviation of the ELISA technical replicates.

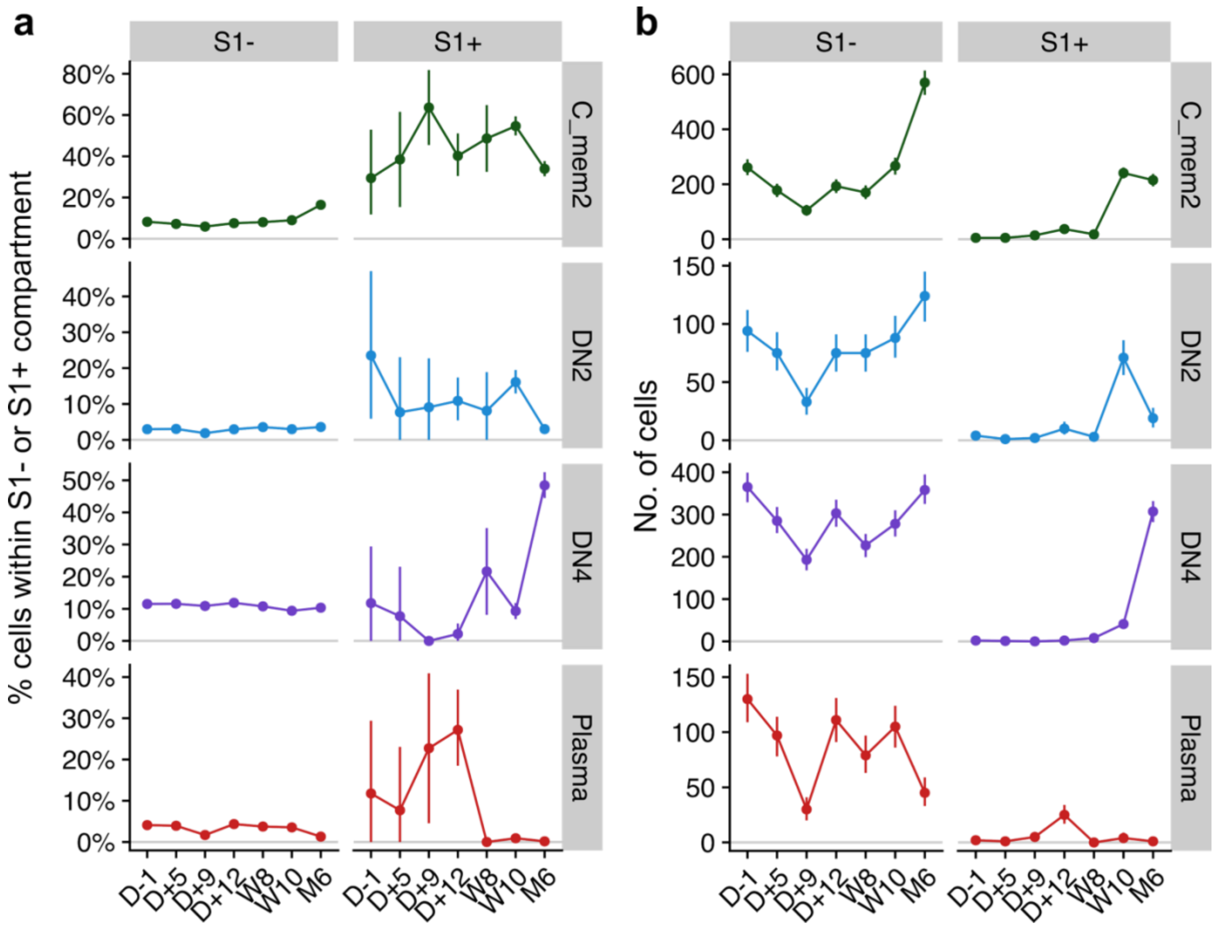

Figure S5. Bootstrapped sampling of relative and absolute cell counts in scRNA-seq data. Related to Figure 1.

(a-b) Bootstrapped sampling of (a) the relative proportion of B cell subsets, and (b) absolute cell numbers, within the $S1^-$ or $S1^+$ compartment in the scRNA-seq data collected in this study. Error bars denote the 95% confidence intervals obtained via bootstrapping conducted separately for each time point. Cell subpopulations discussed in the main text were included here (n=5).

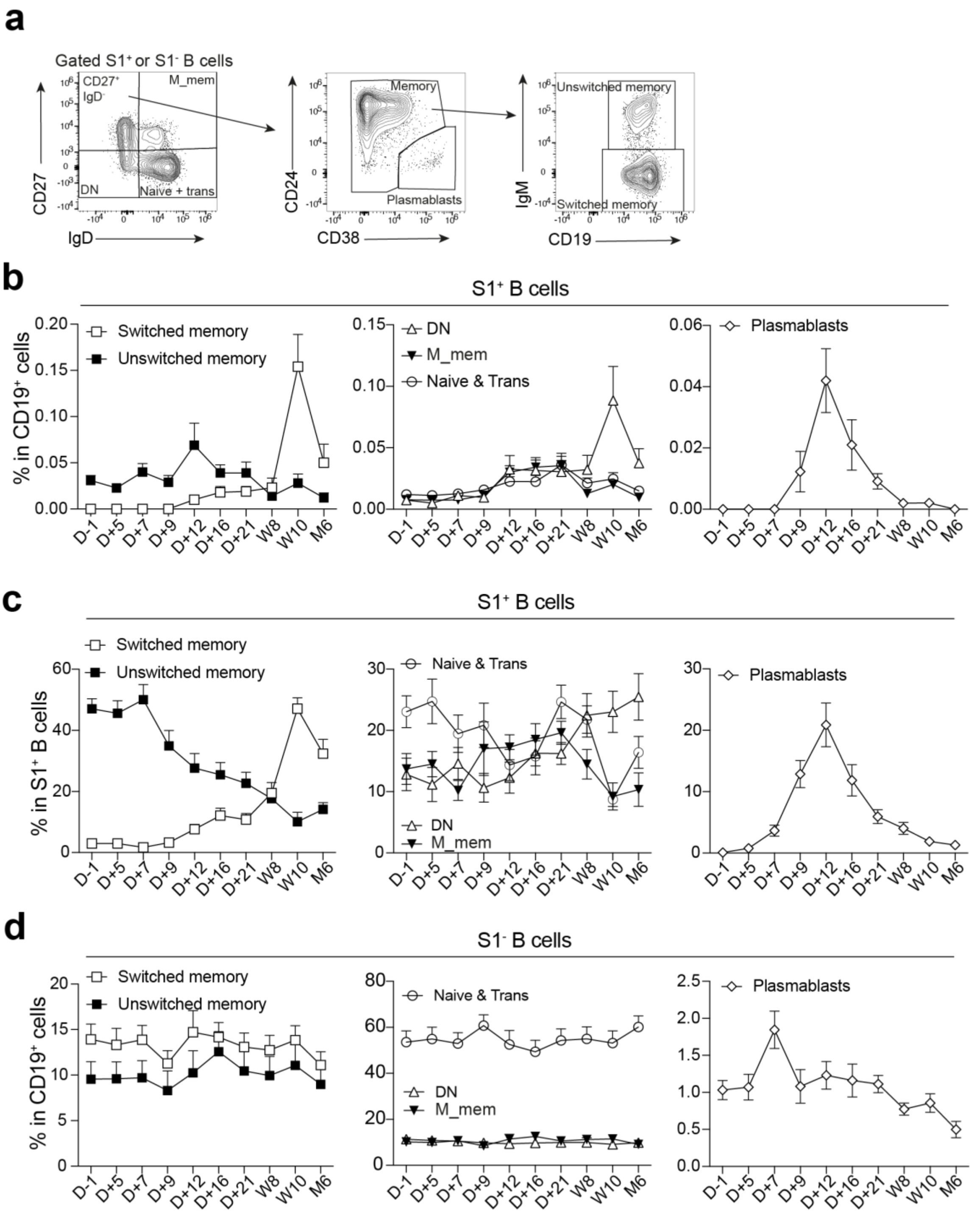

Figure S6. S1⁺ and S1⁻ B cell immunophenotyping. Related to Figure 1.

(a) Gating strategy for identification of vaccine-derived antigen-specific (S1⁺) or general B cell population (S1⁻) class-switched memory B cells (CD19⁺CD27⁺IgD⁻IgM⁻), unswitched memory B cells (CD19⁺CD27⁺IgD⁻IgM⁺), IgM memory (M_mem) B cells (CD19⁺CD27⁺IgD⁺), double-negative (DN) B cells (CD19⁺CD27⁻IgD⁻), naive plus transitional B cells (CD19⁺CD27⁻IgD⁺), and plasmablasts (CD19⁺CD27⁺IgD⁻CD24⁻CD38⁺).

(b-c) Frequencies of S1⁺ class-switched memory B cells (empty squares), S1⁺ unswitched memory B cells (filled squares), S1⁺ M_mem B cells (inverted filled

triangles), S1$^+$ DN B cells (empty triangles), S1$^+$ naive plus transitional B cells (empty circles), and S1$^+$ plasmablasts (empty diamonds) as (b) percentage of CD19$^+$ cells and (c) percentage of S1$^+$ B cells during vaccine response quantified using flow cytometry data. n=8-12; error-bars depict standard error of means.

(d) Frequencies of S1$^-$ class-switched memory B cells (empty squares), S1$^-$ unswitched memory B cells (filled squares), S1$^-$ M_mem B cells (inverted filled triangles), S1$^-$ DN B cells (empty triangles), S1$^-$ naive plus transitional B cells (empty circles), and S1- plasmablasts (empty diamonds) as percentage of CD19$^+$ cells during vaccine response quantified using flow cytometry data. n=9-12; error-bars depict standard error of means.

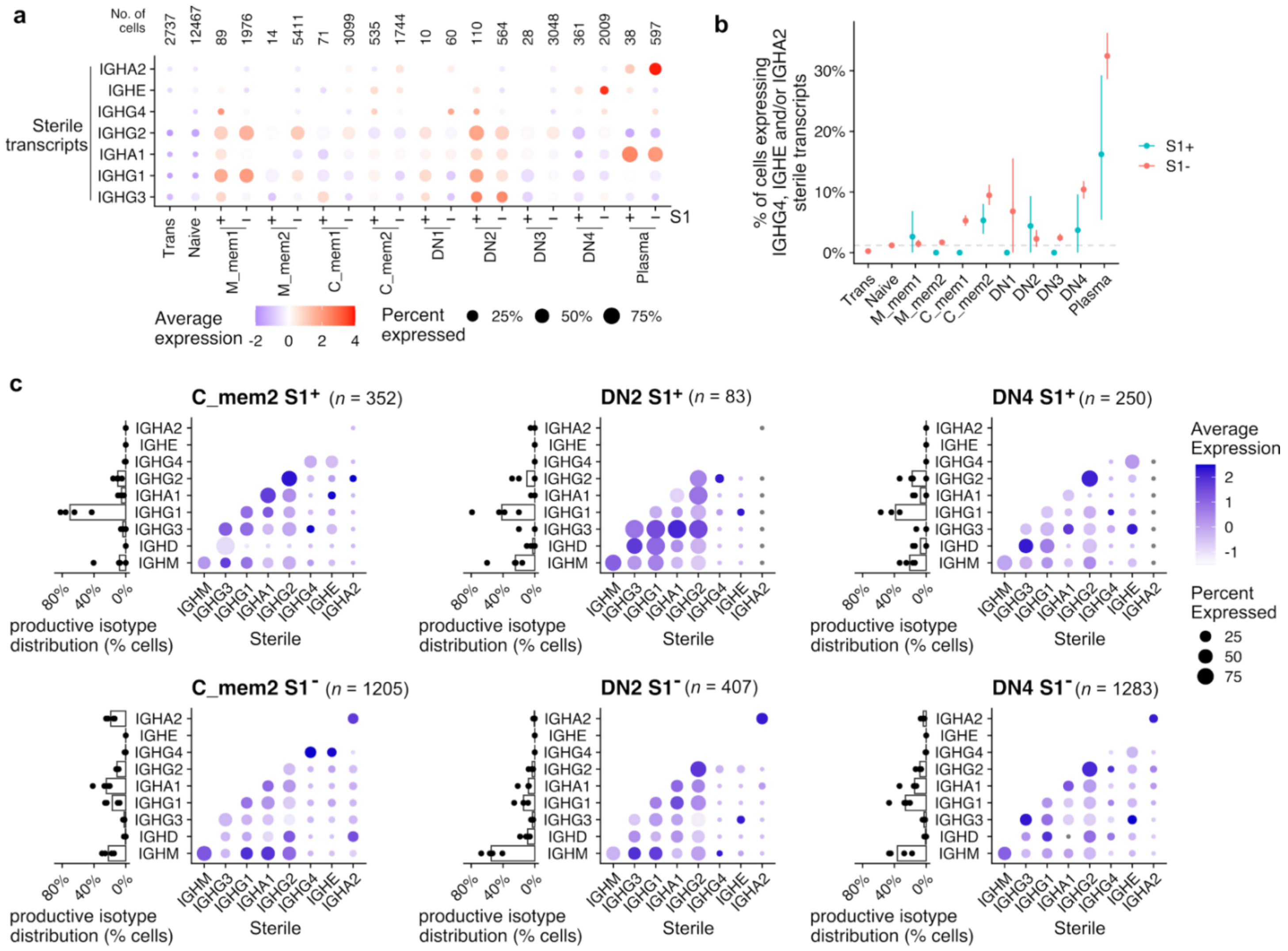

Figure S7. Sterile transcription patterns across cell types and antigen specificity. Related to Figure 2.

(a) Dotplot depicting expression levels of sterile transcripts in our scRNA-seq dataset, as analyzed using sciCSR. B cells were grouped by their B cell subpopulations and into S1- and S1+.

(b) Proportion of cells expressing sterile transcripts of *IGHG4*, *IGHE* and *IGHA2* subtypes in our scRNA-seq dataset grouped by their B cell subpopulations and S1 specificity. Error bars displayed 95% confidence intervals obtained via bootstrapped sampling (n=5).

(c) Quantification separately for $S1^+$ and $S1^-$ cells in the C_mem2, DN2 and DN4 subsets, in terms of their productive BCR isotype distribution (left, bar plot) determined using sc BCR-seq data, and sterile transcription levels for B cells of different BCR isotypes (right, dot plot). For bar plots, data points correspond to individual donors.

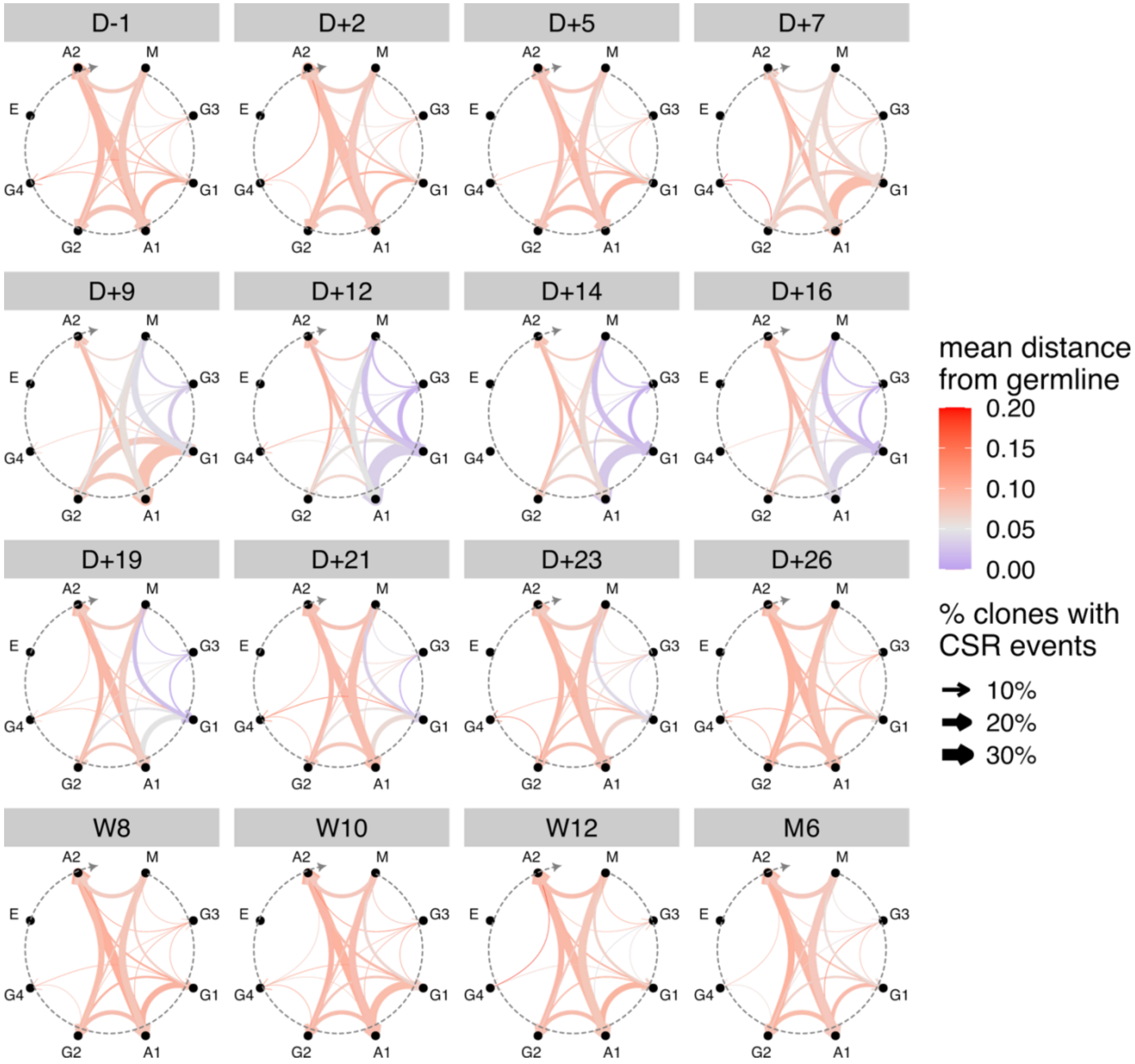

Figure S8. Evolution of CSR patterns sampled in the bulk BCR repertoire dataset across the entire time series. Related to Figure 3.

n=84,855 class-switch events sampled from the bulk BCR repertoire dataset, expressed as a carousel of BCR isotypes arranged clockwise, matching the physical organization of the human *IGHC* gene locus. Arrows connect the start and end points of class-switching, with their width proportional to frequency of class-switch events and color depicting the mutational level at which class-switching was estimated to occur.

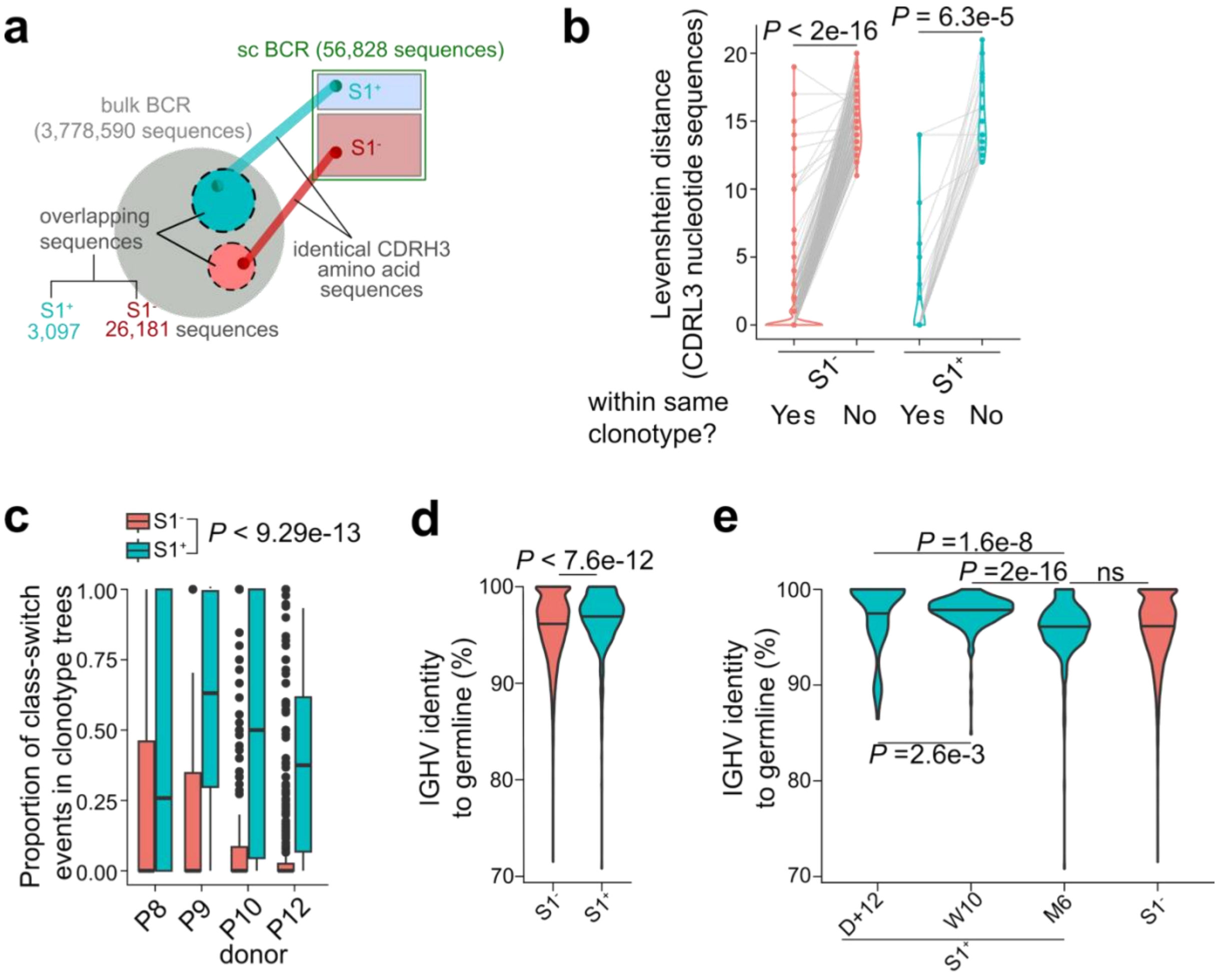

Figure S9. Matching single-cell and bulk BCR repertoires reveal elevated class-switch recombination and low somatic hypermutation as features of S1⁺ B cells of naive individuals after SARS-CoV-2 mRNA vaccination. Related to Figure 3.

(a) Schematic illustrating matching of single-cell and bulk BCR sequences, based on identical CDRH3 amino acid sequences, to define S^+ and S^- BCR sequences sampled in both assays.

(b) For n = 298 clonotypes matched between the bulk and single-cell datasets based on CDRH3 identity, the CDRL3 nucleotide sequences were compared using the Levenshtein distances. Comparisons were made both within and across clonotypes, and separately for S1- and S1+ clonotypes. P-values (Wilcoxon sign-rank test) adjusted using the FDR method were reported.

(c) Comparison of n=1,647 clonotype trees in terms of frequency of class-switch branches connecting sequences with different isotypes. Only donors with at least 10 $S1^+$ clonotype trees were considered in this analysis. Statistical evaluation and derived p-values were obtained using a mixed-effect model with antigen specificity ($S1^+$/$S1^-$) as the fixed effect and donor identifiers as random effects.

(d-e) Comparison of n=19,764 B cells from the scRNA-seq data, excluding Transitional and Naive B cells, in terms of BCR somatic hypermutation (e) between $S1^+$ B cells and $S1^-$ B cells, and (f) specifically of $S1^+$ B cells between D+12, W8 and M6, and

$S1^-$ B cells at all timepoints. Statistical comparisons were based on Wilcoxon's rank-sum test and p-values were corrected for multiple test corrections based on the Benjamini-Hochberg method.

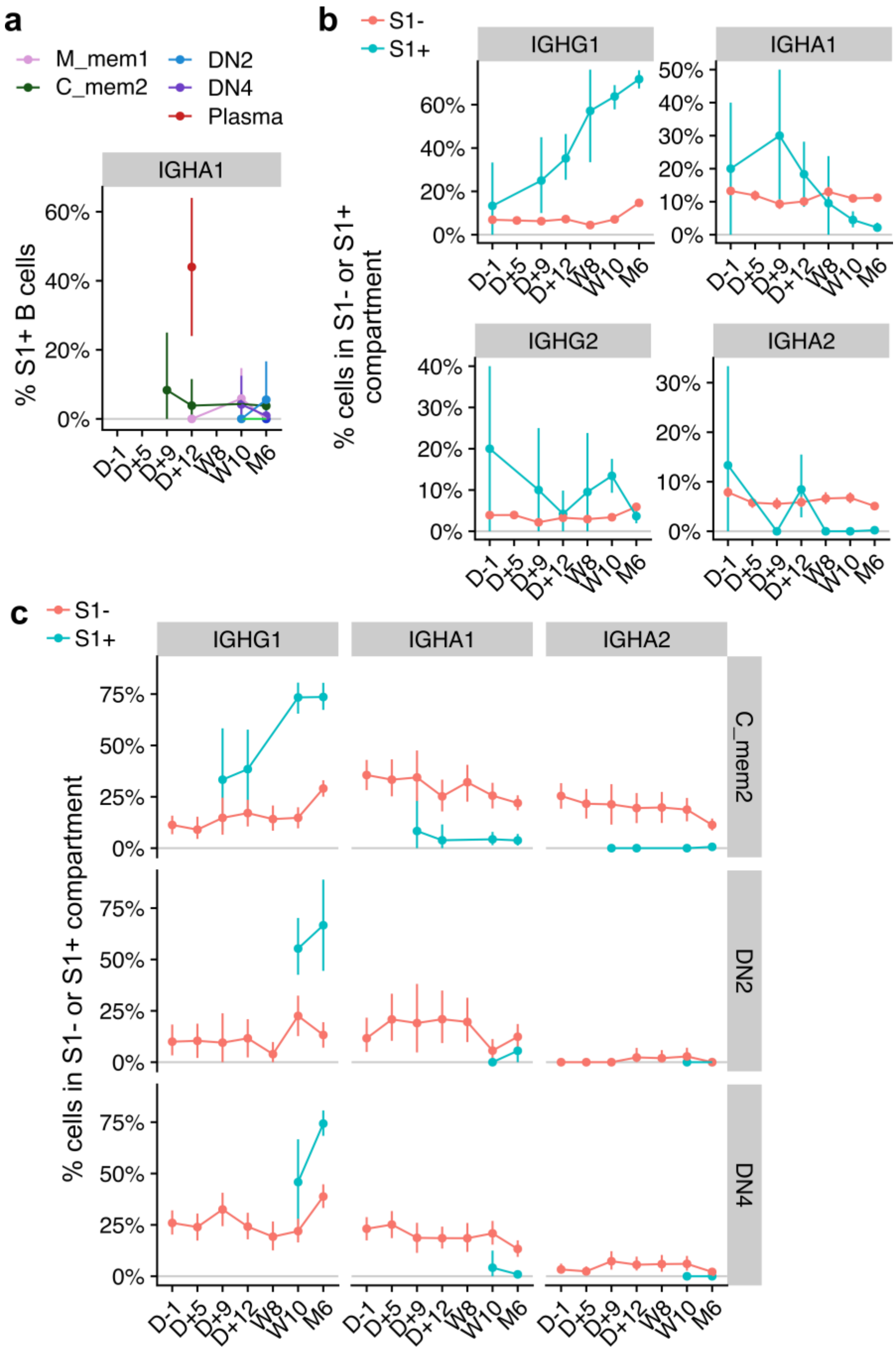

Figure S10. Bootstrapped sampling of BCR isotype distribution in scRNA-seq data. Related to Figure 4.

In panels a to c, error bars denote 95% confidence intervals obtained via bootstrapped sampling. Data were only shown for metadata combinations with at least 10 B cells.

(a) Proportion of $S1^+$ $IGHA1^+$ B cells across time points, with data grouped by B cell subpopulations (n=5).

(b) Proportion of $S1^-$ and $S1^+$ B cells bearing the *IGHG1*, *IGHG2*, *IGHA1* and *IGHA2* isotype in our scRNA-seq dataset across time points (n=5).

(c) Proportion of $S1^-$ and $S1^+$ C_mem2, DN2 and DN4 B cells bearing the *IGHG1*, *IGHA1* and *IGHA2* isotypes in the scRNA-seq data (n=5).

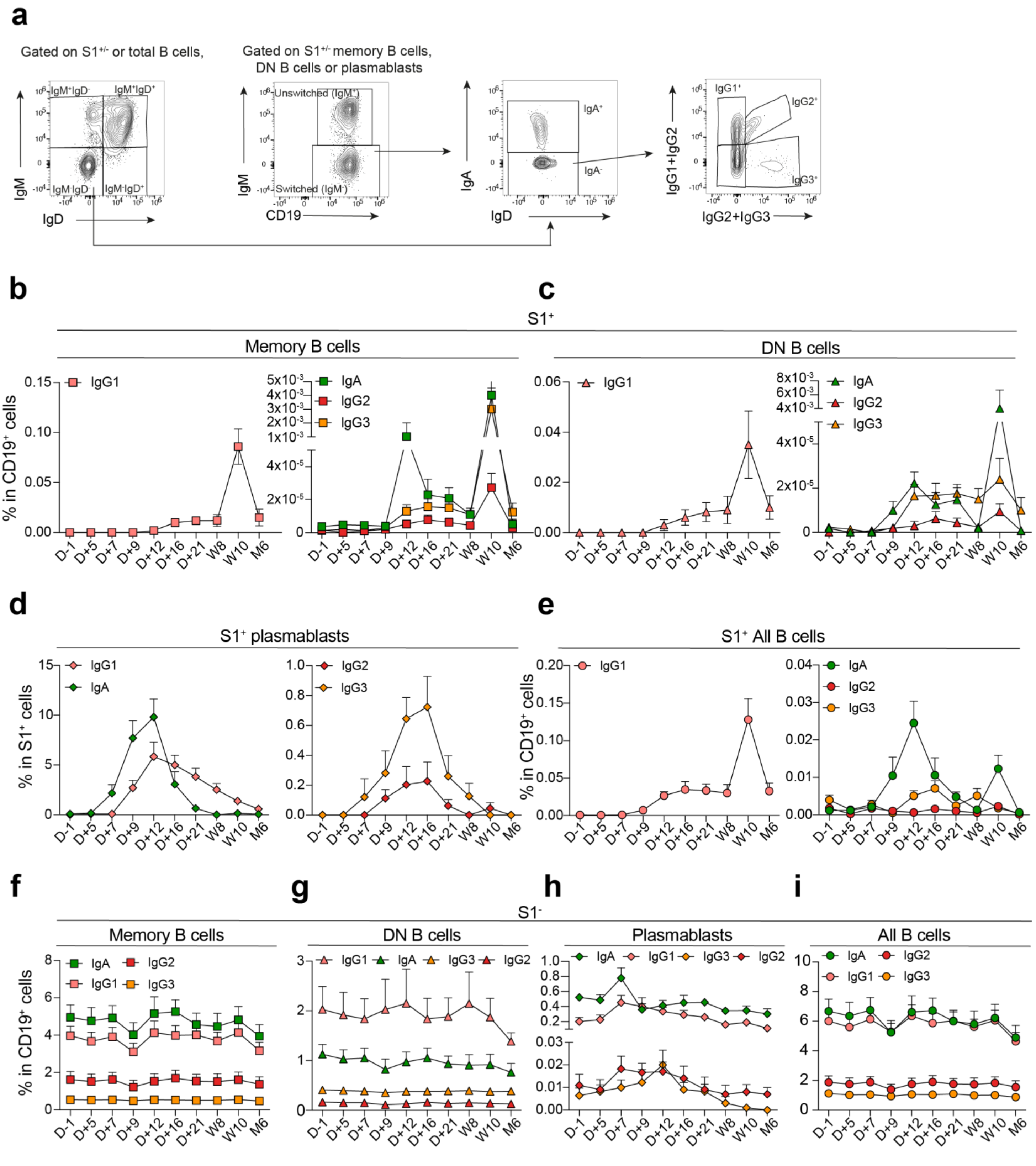

Figure S11. BCR isotype analysis in flow cytometry for S1$^+$ and S1$^-$ B cells. Related to Figure 4.

(a) Gating strategy for identification of IgA$^+$, IgG1$^+$, IgG2$^+$ and IgG3$^+$ class-switched total B cells (CD19$^+$IgD$^-$IgM$^-$) class-switched memory B cells (CD19$^+$CD27$^+$CD24$^+$CD38^{lo}IgD$^-$IgM$^-$), class-switched double-negative (DN) B

cells ($CD19^+CD27^-IgD^-IgM^-$), and class-switched plasmablasts ($CD19^+CD27^+IgD^-CD24^-CD38^+IgD^-IgM^-$) in both vaccine-derived antigen-specific ($S1^+$) or general B cell population ($S1^-$).

(b-e) Frequencies of $S1^+$ class-switched memory B cells ($CD19^+CD27^+IgD^-IgM^-$, squares), class-switched double-negative (DN) B cells ($CD19^+CD27^-IgD^-IgM^-$, triangles), class-switched plasmablasts ($CD19^+CD27^+IgD^-CD24^-CD38^+IgD^-IgM^-$, diamonds) and class-switched total B cells ($CD19^+IgD^-IgM^-$, circles) as percentage of $CD19^+$ or $S1^+$ B cells grouped by BCR isotype quantified using flow cytometry data during vaccines response. n=8-11; error-bars depict standard error of means.

(f-i) Frequencies of $S1^-$ class-switched memory B cells ($CD19^+CD27^+IgD^-IgM^-$, squares), class-switched double-negative (DN) B cells ($CD19^+CD27^-IgD^-IgM^-$, triangles), class-switched plasmablasts ($CD19^+CD27^+IgD^-CD24^-CD38^+IgD^-IgM^-$, diamonds) and class-switched total B cells ($CD19^+IgD^-IgM^-$, circles) as percentage of $CD19^+$ B cells grouped by BCR isotype quantified using flow cytometry data during vaccines response. n=9-11; error-bars depict standard error of means.

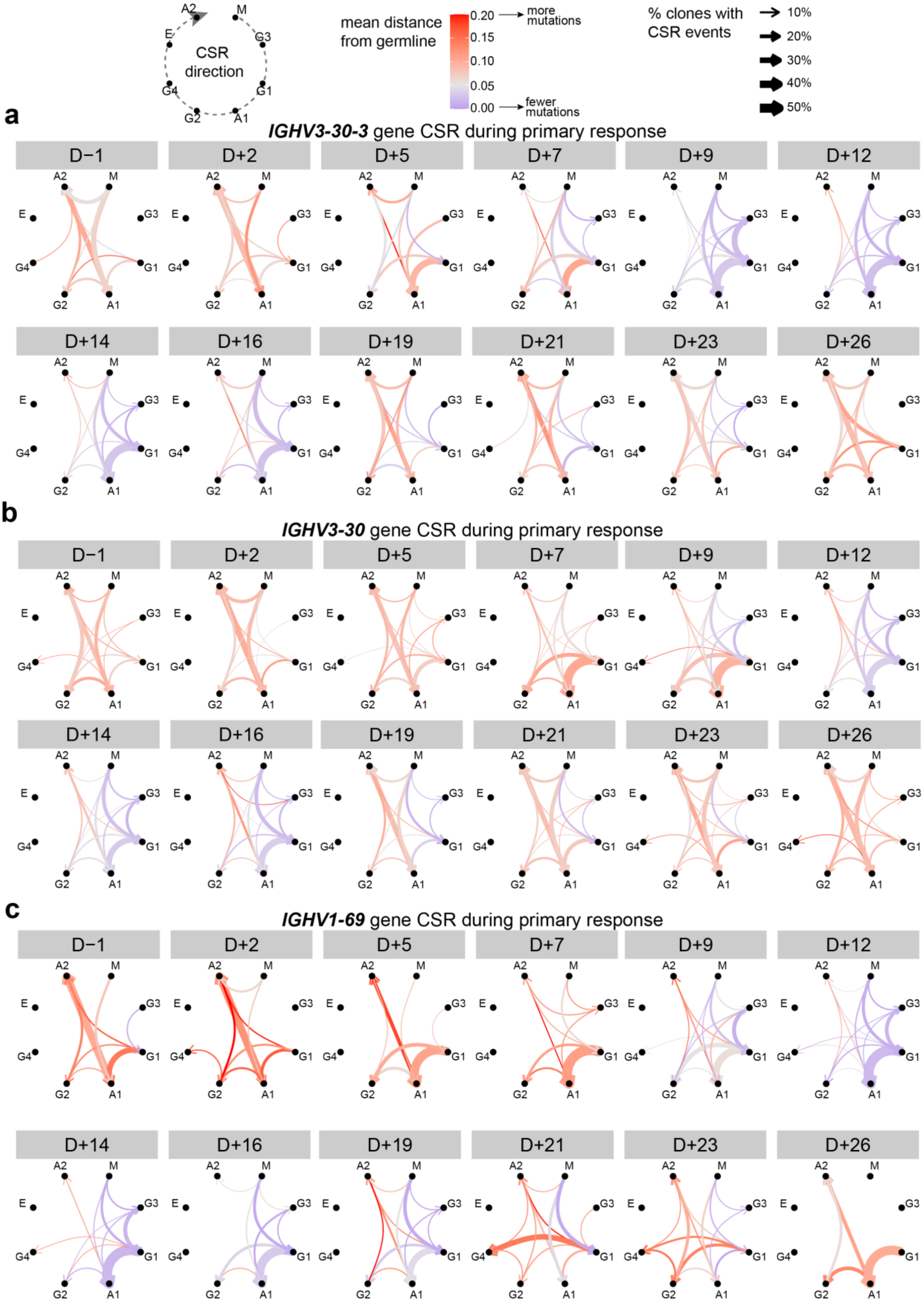

CSR direction
A2
M
G3
G1
A1
G2
G4
E
mean distance from germline
0.20 more mutations
0.15
0.10
0.05
0.00 fewer mutations
% clones with CSR events
10%
20%
30%
40%
50%
a
IGHV3-30-3 gene CSR during primary response
D−1
D+2
D+5
D+7
D+9
D+12
D+14
D+16
D+19
D+21
D+23
D+26
b
IGHV3-30 gene CSR during primary response
D−1
D+2
D+5
D+7
D+9
D+12
D+14
D+16
D+19
D+21
D+23
D+26
c
IGHV1-69 gene CSR during primary response
D−1
D+2
D+5
D+7
D+9
D+12
D+14
D+16
D+19
D+21
D+23
D+26

Figure S12. Evolution of CSR patterns in clonotypes utilizing specific *IGHV* genes sampled in the bulk BCR repertoire dataset. Related to Figure 5.

As in Supplementary Figure 8 but depicting class-switch events from clonotypes utilizing (a) *IGHV3-30-3* (n=765 clonotypes), (b) *IGHV3-30* (n=2,330) and (c) *IGHV1-69* (n=575), expressed as a carousel of BCR isotypes arranged clockwise, matching the physical organization of the human *IGHC* gene locus. Timepoints up to D+26 are depicted. Arrows connect the start and end points of class-switching, with their width proportional to frequency of class-switch events and color depicting the mean mutational level of sequences involved in the given class-switching event.

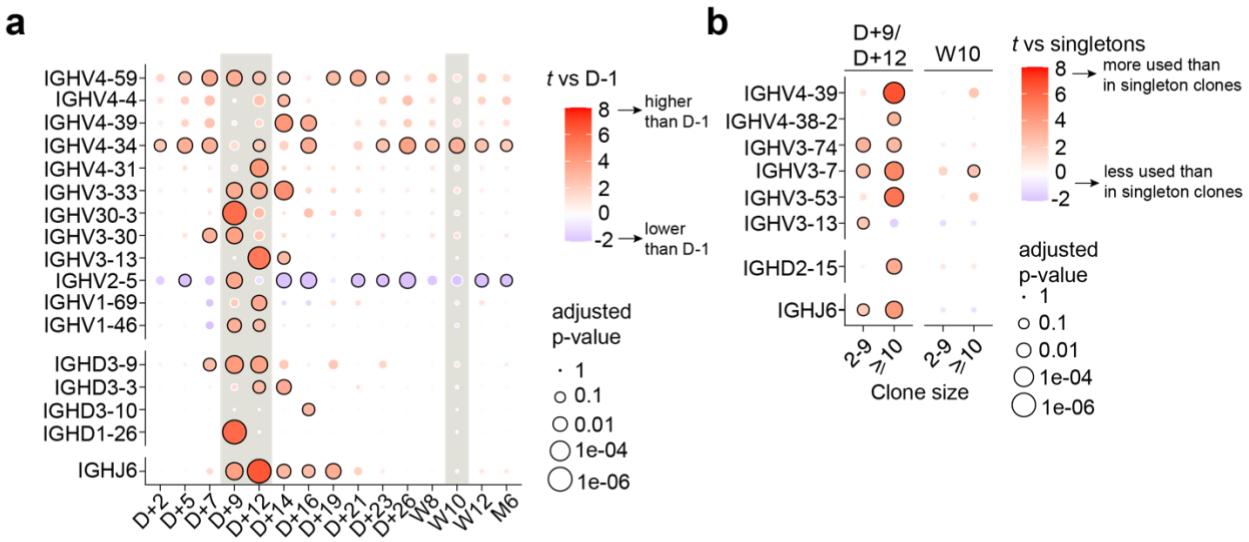

Figure S13. Differential polyclonality in VDJ gene usage during first versus second peak. Related to Figure 5.

(a) Change in immunoglobulin heavy-chain variable (V), diversity (D) and J (joining) gene usage across timepoints in the bulk BCR sequencing data (n=1,511,787 heavy chains). First (D+9 and D+12) and second (W10) response peaks are boxed in grey. Statistical significance was assessed by fitting mixed-effect linear models of percentage gene usage (dependent variable) against time point as the fixed effect and donor identifiers as the random effect. Bubble colors depict effect size compared to D-1 (positive values in red indicate elevated usage of gene compared to D-1), and bubble sizes correspond to p-value after false-discovery rate adjustment.

(b) Comparison of heavy-chain V, D and J gene usage between clonotypes of different sizes. Gene usage was computed for sequence subsets as defined in (a), separately for time points at the first peak (D+9 and D+12) and the second peak (W10). Clonotypes were grouped by their sizes, into singletons, clonotypes with 2-9 sequences and those with more than 10 sequences (“>=10”). Mixed effect models were fitted to compare gene usage against singleton clonotypes as a control, with clonotype sizes as fixed effects and donors as random effects. Bubble colors depict effect size (positive values in red indicate elevated usage of gene), and bubble sizes correspond to p-value after false-discovery rate adjustment.

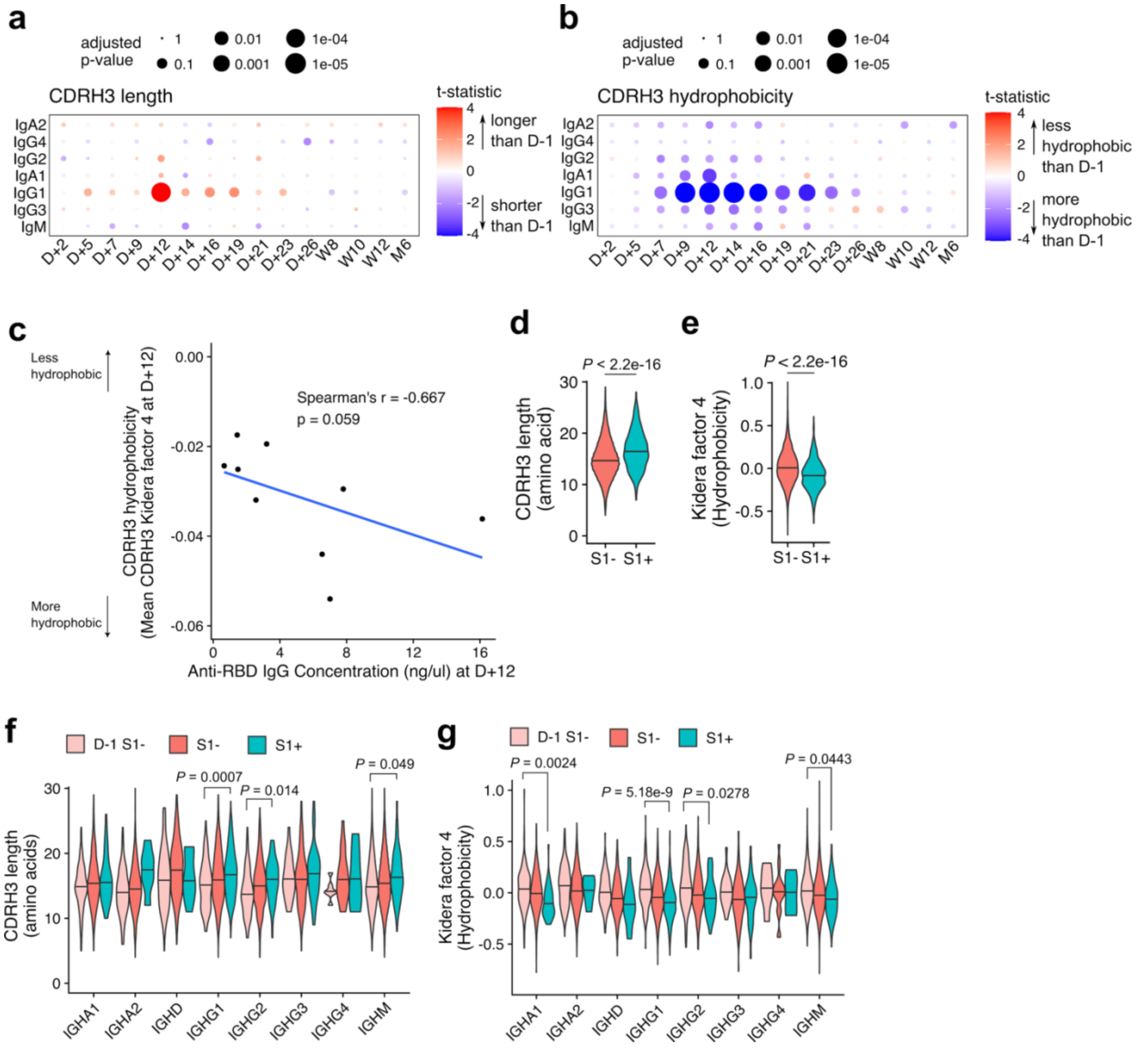

Figure S14. Longer and more hydrophobic CDRH3 is a feature of the vaccine-induced primary antibody response. Related to Figure 5.

(a-b) Comparison of CDRH3 (a) amino acid sequence length and (a) hydrophobicity (using the Kidera factor 4 [Kidera et al. 1985] as a proxy) across time points. Sequences (n=1,511,787) from the bulk BCR sequencing data were separated by isotypes and separate mixed effect models were fitted for CDRH3 length and Kidera factor 4 to compare each time point against D-1 as control, with donors as random effects. Bubble color indicates t-statistic from the mixed effect models, whilst bubble size indicates p-value after false-discovery rate correction.

(c) Association between Anti-RBD IgG concentration as measured using ELISA (horizontal axis) and mean CDRH3 Kidera Factor 4 (indicating hydrophobicity) measured using D+12 sera from n=9 donors.

(d-e) CDRH3 (d) amino acid sequence length and (e) Kidera factor 4 hydrophobicity distributions for $S1^+$ (n=1,133) and $S1^-$ (n=16,712) B cells, subsetting for cells other than naive and transitional B cells. Statistical comparison was performed

using a Wilcoxon rank-sum test.

(f-g) Comparison of CDRH3 (f) amino acid length and (g) hydrophobicity (using the Kidera factor 4 [Kidera et al. 1985] as a proxy) between $S1^{+}$ (n=1,187) and $S1^{-}$ (n=27,806) B cells profiled in the scBCR-seq data. Distributions were visualized as violin plots separately for different BCR isotypes. $S1^{-}$ data from D–1 (n=4,560) was visualized separately to represent the baseline. Statistical comparisons of the D-1 $S1^{-}$ baseline against $S1^{+}$ were computed using Wilcoxon rank-sum tests and *p*-values were adjusted for multiple test corrections using the Benjamini-Hochberg method.

Participant ID	Sex	Age	CMV status
P01	F	34	IgG Negative
P02	F	24	IgG Positive
P03	M	32	IgG Positive
P04	M	24	IgG Positive
P05	M	28	IgG Negative
P06	F	30	IgG Negative
P07	F	29	IgG Positive
P08	F	29	IgG Positive
P09	F	28	IgG Positive
P10	F	29	IgG Positive
P11	F	29	IgG Positive
P12	F	27	IgG Negative
P13	F	29	IgG Positive
P14	M	35	IgG Positive
P15	F	24	IgG Negative
Average/ratio	73% F	28.7	66% IgG positive

Table S1: participants demographic details. Related to STAR Methods. CMV: human cytomegalovirus M:male, F:female.

B cell cluster designation	B cells cluster full name	Gene signature
Plasma	Plasmablasts	CD27, CD38, PRMD1, XBP1, JCHAIN
DN4	Double negative 4	SELL, CD53, HOPX, IGE, IL13RA1
DN3	Double negative 3	FOS, CXCR4, CD83, CD69
DN2	Double negative 2	TBX21, ZEB2, FCRL5
DN1	Double negative 1	PPP1R14A, CRIP1, CRIP2, S100A10, TAGLN2 ANXA2
C_mem2	Classical memory 2	SELL, CD53, ACTB, HOPX, CRIP1, CRIP2, S100A10, TAGLN2, ANXA2, ANXA4
C_mem1	Classical memory 1	CD24
M_mem1	IgM memory 1	IGM, SELL, AP3B1, CXCR4
M_mem2	IgM memory 2	IGM, CD1C, CD24
Naive	Mature naive	IGD, IGM, TCL1A, FCER2, SELL, IL4R, CCR7 CXCR4
Trans	Transitional	FOS, CD9, IGD, IGM, TCL1A, CXCR4

Table S2: definition of B cell subsets based on differentially expressed genes in Supp. Fig 2f.
